# From Ethnomedicine to Pharmacological Evidence: Phytochemistry, Biological Activities, and Therapeutic Potential of the Genus *Abutilon* (Malvaceae)

**DOI:** 10.3390/ijms27219768

**Published:** 2026-10-30

**Authors:** Nurgali Rakhymbayev, Galiya Ibadullayeva, Serzhan Mombekov, Aigerim Kantureyeva, Balzhan Azimkhanova, Meruyert Amantayeva, Sabit Pazilov, Ulzhan Iskakova, Nyshanbay Konash, Daryono Hadi Tjahjono, Akdidar Yegizbayeva, Kairat Zhakipbekov, Arailym Daulbayeva

**Affiliations:** 1 Faculty of Pharmacy, Joint-Stock Company “ South Kazakhstan Medical Academy”, Al-Farabi Square, 1, 160000 Shymkent, Kazakhstan; nurgalirakhymbayev@gmail.com (N.R.); arai_14@bk.ru (A.D.); 2 School of Pharmacy, Asfendiyarov Kazakh National Medical University, Tole Bi 94, 050012 Almaty, Kazakhstan; ibadullayeva.g@kaznmu.kz (G.I.); mombekov.s@kaznmu.kz (S.M.); kantureeva.a@kaznmu.kz (A.K.); konashnyshan@gmail.com (N.K.); 3 Department of Biochemistry, Asfendiyarov Kazakh National Medical University, Tole Bi 94, 050012 Almaty, Kazakhstan; azimkhanova.b@kaznmu.kz; 4 Karatai Turysov Institute of Geology and Oil and Gas Engineering, Satbayev University, 22 Satbaev Str., 050013 Almaty, Kazakhstan; m.amantayeva@satbayev.university; 5 City Polyclinic No. 10, 17 Aksai-4 Microdistrict, 050063 Almaty, Kazakhstan; sabit75_p@mail.ru; 6 Department of Microbiology and Virology, Asfendiyarov Kazakh National Medical University, Tole Bi 94, 050012 Almaty, Kazakhstan; iskakova.u@kaznmu.kz; 7 School of Pharmacy, Bandung Institute of Technology, Bandung 40132, Indonesia; daryonohadi@itb.ac.id

**Keywords:** *Abutilon*, Malvaceae, phytochemistry, ethnomedicine, pharmacological activity, molecular mechanisms, toxicology, pharmaceutical translation

## Abstract

The genus *Abutilon* (Malvaceae) comprises taxonomically and phytochemically diverse medicinal plants with a long history of ethnomedicinal use. This critical narrative review integrates evidence on the taxonomy, traditional applications, phytochemical composition, pharmacological activities, molecular mechanisms, safety, and pharmaceutical potential of *Abutilon* species, incorporating the literature published through September 2026. Flavonoids, phenolic acids, triterpenoids, phytosterols, polysaccharides, and fatty acids represent major reported constituent classes, although the reliability of their identification and quantitative characterization varies among studies. *A. indicum* and *A. theophrasti* remain the most extensively investigated species. Experimental findings support preliminary antioxidant, anti-inflammatory, antimicrobial, antidiabetic, hepatoprotective, gastroprotective, and other biological activities, predominantly in biochemical, cellular, and animal models. Mechanistic investigations have reported modulation of NF-κB/MAPK signaling, inflammatory mediators, glucose-regulatory processes, and oxidative stress-related responses; however, direct molecular target validation remains limited. Pharmaceutical studies have explored tablets, phytosomal formulations, and plant-mediated nanoparticles, but evidence of improved systemic bioavailability, therapeutic efficacy, and long-term safety remains insufficient. Toxicological findings indicate preparation- and dose-dependent responses, while controlled clinical evidence is extremely limited. Methodological heterogeneity, incomplete chemical standardization, and limited pharmacokinetic characterization further restrict cross-study comparisons and therapeutic translation. Future research should prioritize authenticated botanical materials, chemically standardized preparations, reproducible dose–response studies, direct mechanistic validation, comprehensive toxicological assessment, and appropriately designed clinical investigations. Overall, *Abutilon* represents a potentially valuable source of pharmacologically active natural products, but current evidence does not establish standardized clinical applications.

## 1. Introduction

Growing interest in multi-target therapeutic strategies, particularly for complex and drug-resistant diseases, has increased attention to natural products capable of modulating multiple biological pathways [1]. Medicinal plants and their bioactive constituents remain important sources of pharmacologically active compounds and potential therapeutic agents. Advances in phytochemistry, pharmacology, and drug-delivery technologies have further expanded opportunities for the investigation and pharmaceutical development of plant-derived compounds [2,3,4].

The genus *Abutilon* (Malvaceae) is widely distributed across tropical and subtropical regions and comprises medicinal plants with considerable taxonomic and phytochemical diversity. Flavonoids, phenolic acids, triterpenoids, phytosterols, and polysaccharides have been reported in several species. Pharmacological investigations have described antioxidant, anti-inflammatory, antimicrobial, antidiabetic, and cytotoxic activities in extracts, fractions, and selected constituents [5,6,7,8]. Related members of Malvaceae, including *Malvastrum* species and *Malva sylvestris*, have also been investigated for their phytochemical composition and biological activities [9,10], providing broader context for research on *Abutilon*.

Species of *Abutilon* have long been used in traditional medical systems, including Ayurveda, Chinese medicine, and regional folk practices. Leaves, roots, seeds, bark, and other plant organs have been employed in preparations intended for inflammatory conditions, infections, gastrointestinal complaints, and skin disorders [11,12]. However, documented traditional use should be distinguished from experimentally demonstrated pharmacological activity and clinically established therapeutic efficacy. In particular, despite the extensive ethnomedicinal history and the growing pharmacological literature concerning *A. theophrasti*, its pharmacokinetic properties remain insufficiently characterized [13]. Despite increasing experimental interest in the genus, the clinical development of Abutilon-derived preparations remains at a preliminary stage. A small, uncontrolled clinical investigation involving *A. indicum* in patients with bronchial asthma has been reported and is critically discussed in Section 7.9. However, the methodological limitations of this investigation preclude definitive conclusions regarding clinical efficacy, and the available evidence does not establish the advancement of Abutilon-derived preparations into adequately controlled clinical development programs.

Previous reviews have summarized the botanical characteristics, ethnomedicinal applications, phytochemistry, and pharmacological properties of *Abutilon* species [5,14]. A comprehensive review incorporating the literature published through 2024 [5] provides an important foundation for understanding the genus. However, a critical integration of experimental evidence remains necessary, particularly regarding the reliability of phytochemical identification, quantitative pharmacological outcomes, experimentally supported molecular mechanisms, toxicological limitations, and barriers to pharmaceutical and clinical translation. These challenges reflect broader limitations in natural-product drug development, including incomplete mechanistic validation, poor or insufficiently characterized bioavailability, limitations of preclinical models, and inadequate clinical evidence [15].

Interpretation of the available *Abutilon* literature is further complicated by substantial methodological heterogeneity. Studies differ in botanical species and plant organs, geographical origin, extraction procedures, chemical characterization, administered concentrations and doses, experimental models, and reference controls. Consequently, results obtained from different investigations cannot always be compared directly, and biological activity observed in preliminary assays does not necessarily predict therapeutic efficacy. Distinguishing chemically characterized and experimentally validated findings from exploratory screening and computational predictions is therefore essential for identifying the most promising research directions.

Accordingly, the present critical narrative review integrates the literature available through September 2026 on the taxonomy, ethnomedicinal uses, phytochemical composition, pharmacological activities, molecular mechanisms, safety, and pharmaceutical potential of *Abutilon*. Particular emphasis is placed on evaluating the strength and limitations of experimental evidence, distinguishing direct molecular validation from pathway-associated or computational findings, and assessing the requirements for chemical standardization, pharmacokinetic characterization, and clinical translation. Rather than repeating existing descriptive reviews, this work aims to identify evidence-supported phytochemical–pharmacological relationships, clarify unresolved methodological and mechanistic questions, and establish priorities for future pharmaceutical investigation.

## 2. Methodology

### 2.1. Review Design and Literature Search Strategy

This study was designed as a critical narrative review to integrate and critically evaluate the available evidence on the taxonomy, ethnomedicinal applications, phytochemical composition, pharmacological activities, molecular mechanisms, safety, and pharmaceutical potential of the genus *Abutilon* (Malvaceae). Particular emphasis was placed on the strength of experimental evidence, phytochemical–pharmacological relationships, methodological heterogeneity, and barriers to pharmaceutical and clinical translation.

The literature was collected and evaluated over approximately one year of manuscript preparation. Searches were conducted using Scopus, Web of Science, PubMed, and Google Scholar, with the final search performed in September 2026. Plants of the World Online (POWO) and World Flora Online (WFO) were additionally consulted to assess current taxonomic nomenclature and geographical distribution, with POWO used as the principal reference for accepted species names within the genus. Mendeley reference management software (version 2.149.0; Elsevier, Amsterdam, The Netherlands) was used during manuscript preparation to organize retrieved publications and manage bibliographic records.

The search combined the term “*Abutilon*” with individual species names, including “*Abutilon indicum*”, “*Abutilon theophrasti*”, “*Abutilon pannosum*”, “*Abutilon fruticosum*”, “*Abutilon bidentatum*”, “*Abutilon grandiflorum*”, and “*Abutilon hirtum*”. These terms were combined, where appropriate, with keywords related to “phytochemistry”, “chemical constituents”, “ethnomedicine”, “traditional use”, “pharmacology”, “biological activity”, “molecular mechanism”, “molecular target”, “pharmacognosy”, “toxicity”, “safety”, “pharmaceutical formulation”, and “drug delivery”.

The literature search primarily covered peer-reviewed publications published between 2000 and September 2026. Earlier studies were considered when they provided original or foundational evidence concerning phytochemical constituents, ethnomedicinal applications, pharmacognostic characteristics, or pharmacological effects not adequately represented in more recent publications. Reference lists of relevant original studies and review articles were additionally examined to identify potentially relevant publications.

The review aimed to provide broad coverage of the available literature rather than restrict the synthesis to a small number of selected experimental investigations. Nevertheless, the inclusion of publications was guided by their relevance, methodological information, and contribution to the objectives of the review.

### 2.2. Literature Selection and Eligibility Criteria

Publications were selected according to their relevance to the objectives of the review and the availability of sufficient information for scientific interpretation. Studies addressing the taxonomy, geographical distribution, ethnomedicinal uses, phytochemical composition, pharmacognostic characteristics, pharmacological activities, molecular mechanisms, safety, or pharmaceutical development of *Abutilon* species were considered for inclusion.

Literature identification and selection were conducted progressively throughout manuscript preparation. Retrieved publications were assessed for topical relevance, and potentially relevant full-text articles were examined when accessible. Priority was given to original experimental studies reporting identifiable botanical materials or isolated constituents, extraction procedures, experimental models, tested concentrations or doses, and measurable biological outcomes.

Duplicate records, conference abstracts, non-peer-reviewed publications, studies unrelated to the scope of the review, and reports lacking sufficient methodological or experimental information for meaningful interpretation were generally excluded. Review articles were used primarily to provide background context and identify relevant primary studies, whereas conclusions regarding specific experimental findings were preferentially supported by original research publications.

Studies were not excluded solely because they reported weak, inconclusive, or negative pharmacological outcomes. Such findings were considered relevant when they contributed to a balanced interpretation of the available evidence. Similarly, studies involving preliminary screening methods were considered where appropriate, but their methodological limitations were explicitly acknowledged.

The selection of species for detailed discussion was guided by the availability, relevance, and methodological adequacy of published evidence. Consequently, the review does not claim exhaustive experimental coverage of every species currently recognized within the genus *Abutilon*.

### 2.3. Evidence Classification and Critical Appraisal

To distinguish the nature and strength of the available evidence, pharmacological findings were categorized according to their experimental context: chemical or biochemical assays, in vitro cellular studies, ex vivo investigations, in vivo animal experiments, formulation studies, and clinical investigations. Ethnomedicinal reports were considered separately as documentation of traditional applications rather than direct evidence of therapeutic efficacy. These categories were used to describe the type and translational relevance of evidence and did not constitute a formally validated risk-of-bias or quantitative quality-scoring system.

For each pharmacological study, particular attention was given to the investigated species, plant part, extraction solvent or preparation, chemical characterization, experimental model, dose or concentration, reference controls, and reported biological outcomes. Where sufficient information was available, these parameters were used to evaluate consistency, reproducibility, and the limitations of comparisons across studies.

Phytochemical findings were interpreted according to the analytical methods used for compound identification. Preliminary chromatographic screening and tentative identifications based primarily on mass-spectral or database matching were distinguished from compounds identified using authentic reference standards or supported by isolation and structural characterization through complementary spectroscopic techniques, including nuclear magnetic resonance spectroscopy.

Mechanistic evidence was evaluated by distinguishing changes in molecular markers and signaling pathways from experimentally demonstrated causal mechanisms or direct molecular target engagement. Computational predictions, including molecular docking, were considered hypothesis-generating unless supported by appropriate biochemical, cellular, or functional validation.

Chemical antioxidant assays, including DPPH, ABTS, and FRAP, were interpreted as measures of radical-scavenging or reducing capacity rather than direct evidence of therapeutic antioxidant efficacy. Similarly, antimicrobial activity, cytotoxicity, and other pharmacological findings were interpreted in relation to their experimental models, tested concentrations, reference controls, and available evidence of biological selectivity.

The strength of evidence was assessed qualitatively by considering the biological relevance of the experimental model, the availability of quantitative concentration–response data, the use of appropriate reference treatments, the extent of chemical characterization, and the presence of complementary mechanistic or functional outcomes. Findings supported by multiple experimental approaches were generally considered more informative than isolated screening results, while differences in study design and methodological quality were taken into account when interpreting apparently consistent or contradictory observations.

### 2.4. Qualitative Evidence Synthesis and Reporting Limitations

The selected literature was organized into thematic domains covering ethnomedicinal uses, phytochemistry, pharmacological activities, molecular mechanisms, pharmaceutical applications, and safety. A qualitative critical synthesis was undertaken to identify recurring findings, differences among experimental systems, contradictory observations, and limitations affecting the interpretation and translational relevance of the available evidence.

Particular attention was given to distinguishing preliminary screening results from findings supported by complementary cellular, animal, mechanistic, or clinical evidence. Comparisons of numerical outcomes were interpreted cautiously because differences in botanical materials, extraction procedures, chemical standardization, experimental models, concentrations, doses, and reference controls frequently precluded direct comparison.

The review incorporated a broad range of published evidence, including foundational studies and more recent experimental investigations. However, the number of references included in the final manuscript should not be interpreted as evidence that all potentially relevant publications were identified or that the review constitutes an exhaustive systematic synthesis.

This review was not designed or conducted as a PRISMA-based systematic review. Literature identification and selection were performed progressively, and the numbers of initially retrieved, screened, and excluded records were not prospectively documented. Therefore, a reliable PRISMA flow diagram could not be reconstructed, and no formal quantitative meta-analysis was undertaken.

These methodological limitations may introduce selection bias and restrict the reproducibility and completeness of literature identification. Accordingly, the conclusions of this review are presented as a qualitative critical assessment of the available evidence rather than an exhaustive systematic evaluation of all published studies. The principal research gaps and priorities for future pharmacological and pharmaceutical investigations were identified on the basis of the consistency, experimental relevance, and limitations of the available findings.

## 3. Taxonomy and Distribution of the Genus *Abutilon*

### 3.1. Systematic Status and Taxonomic Classification

**Figure 1:**
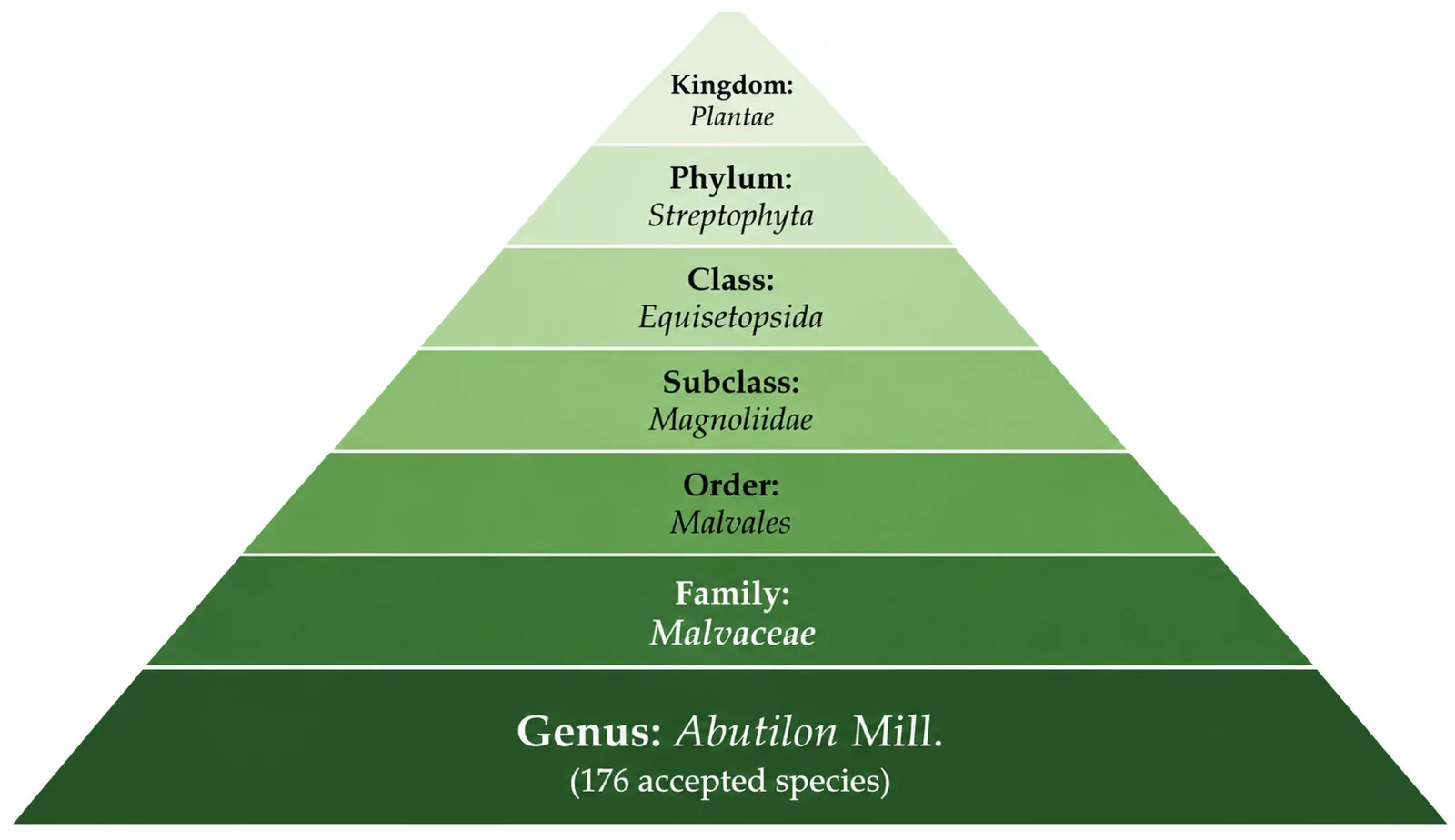
Taxonomic classification of the genus *Abutilon*. The genus comprised 176 accepted species in the Plants of the World Online (POWO) taxonomic backbone consulted during manuscript preparation [20].

The earlier botanical literature estimated that the genus *Abutilon* comprises approximately 150–200 species [16]. Phylogenetic and molecular investigations have contributed to the classification of Malvaceae and improved understanding of the relationships among *Abutilon* and related malvaceous taxa [17,18,19]. According to the Plants of the World Online (POWO) taxonomic backbone consulted during manuscript preparation, 176 species were recognized as accepted within the genus [20]. However, accepted species numbers and taxonomic circumscriptions may change as nomenclatural and phylogenetic evidence is updated. The taxonomic classification adopted for the genus is summarized in Figure 1.

### 3.2. Geographic Distribution and Ecology

**Figure 2:**
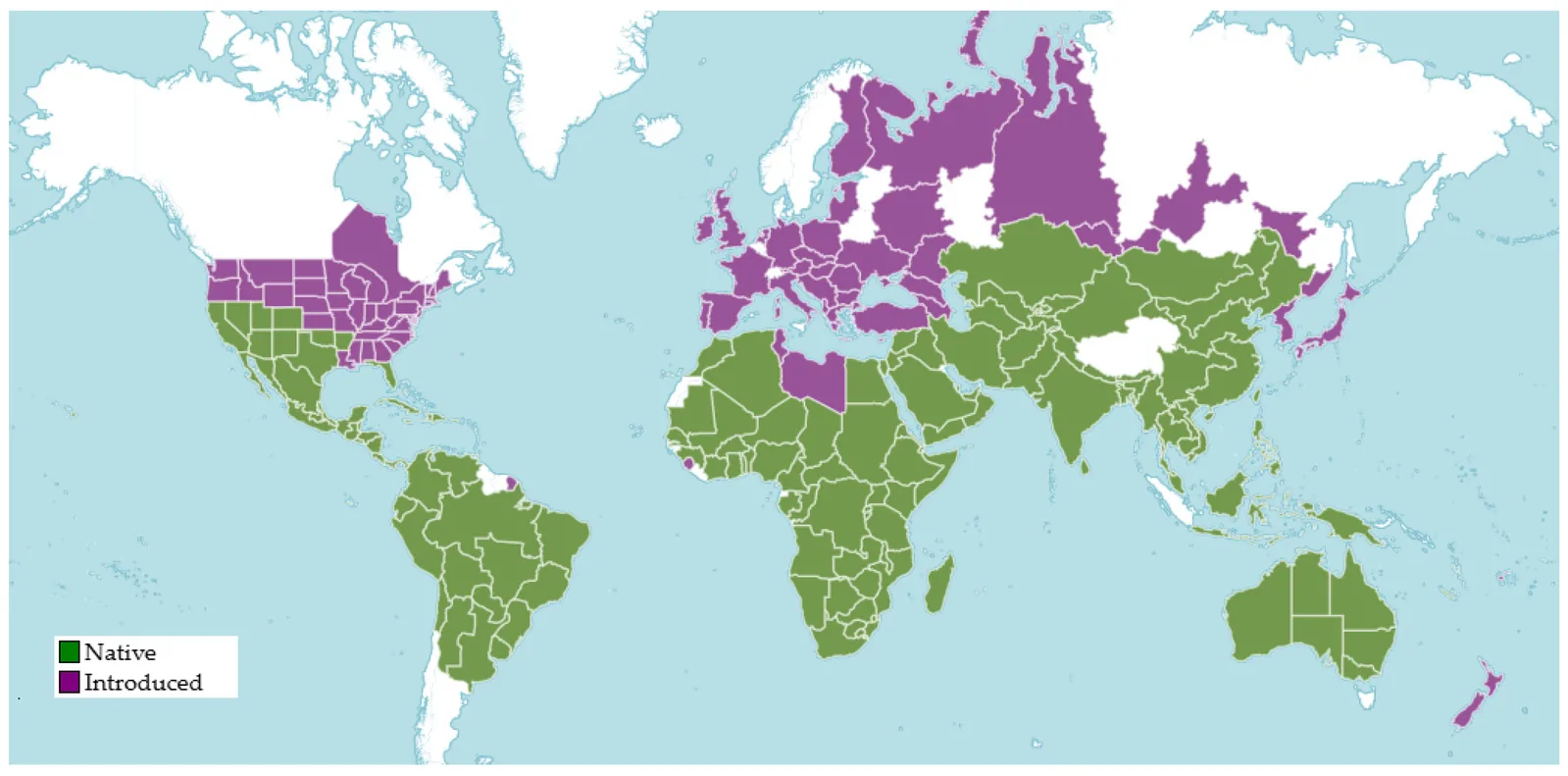
Global native and introduced distribution of the genus *Abutilon* according to Plants of the World Online (POWO), Royal Botanic Gardens, Kew. Green indicates native distribution, whereas purple indicates introduced distribution [20].

Species of *Abutilon* occur widely across tropical and subtropical regions, including the Americas, Africa, Asia, Australia, Oceania, and parts of southern Europe. Their geographical distribution reflects considerable ecological diversity and variation in habitat preferences. Phenotypic plasticity and developmental responses to environmental conditions have been investigated experimentally in *A. theophrasti* [21], while a recent taxonomic revision has documented the diversity of *Abutilon* species in India [22]. The global native and introduced distribution of the genus, as documented by POWO, is illustrated in Figure 2.

The geographical ranges of individual *Abutilon* species continue to be refined through regional floristic investigations. For example, *A. bidentatum* has been reported as an addition to the flora of South India [23]. Several species also occur in disturbed or human-modified habitats. *A. theophrasti*, an annual species frequently associated with cultivated fields, has received considerable attention because of its ecological adaptability and agricultural importance as a weed [24]. Taxonomic investigations have additionally documented morphological variation among members of the genus [25].

The distribution of *A. grandifolium*, a shrubby species, has been documented beyond parts of its native range [26], including a more recent record from Algeria [27]. Such findings demonstrate the importance of distinguishing native occurrence from introduced or newly documented regional populations when interpreting geographical distributions.

Among the species reviewed, *A. indicum* (L.) Sweet is widely distributed across tropical and subtropical Asia, including India, Pakistan, Sri Lanka, and Southeast Asia [28,29]. Its traditional medicinal applications and diverse reported biological activities have contributed to extensive pharmacognostic and pharmacological investigation [30]. The geographical distribution of *A. pannosum* has also been documented through regional floristic records, including reports from India [31].

Other members of the genus remain comparatively less extensively investigated. Pharmacognostic characterization of *A. hirtum* has documented morphological and anatomical features of its leaves, stems, roots, and flowers [32]. Broader reviews have additionally summarized the medicinal importance of selected underexplored *Abutilon* species [33], while phytochemical investigation of *A. bidentatum* has led to the isolation of abubidentin A, an oleanane-type triterpene ester with reported biological activity [34].

Overall, the genus *Abutilon* exhibits substantial taxonomic, geographical, and ecological diversity, whereas the distribution of pharmacological evidence among species remains uneven. *A. indicum* is particularly well represented in the available phytochemical and pharmacological literature [35,36]. Further investigation of less-studied species, supported by reliable botanical authentication and consistent taxonomic nomenclature, is therefore important for improving the representativeness of research across the genus.

### 3.3. General Morphological and Pharmacognostic Characteristics

Species of *Abutilon* comprise herbs and shrubs exhibiting diagnostically relevant variation in vegetative and reproductive morphology [37]. Leaves are generally simple and petiolate, while leaf shape, venation, and trichome characteristics vary among species and may provide useful features for botanical identification [38,39]. Stems and leaves may bear simple, stellate, or glandular trichomes, whereas the flowers are typically axillary and actinomorphic, with five sepals, five petals, and a characteristic staminal column [32].

Pharmacognostic investigations of *A. indicum* have documented diagnostic macroscopic and microscopic leaf characteristics, including covering and glandular trichomes, stomatal features, vascular tissues, calcium oxalate crystals, and palisade and spongy mesophyll organization [40]. Additional studies have reported quantitative leaf constants, physicochemical parameters, powder-microscopic characteristics, and fluorescence profiles relevant to the authentication and quality assessment of crude botanical materials [41].

In *A. hirtum*, anatomical characteristics of leaves, stems, roots, and floral organs, including epidermal, cortical, vascular, and supporting tissues, have also been described [32]. More recently, pharmacognostic investigation of *A. theophrasti* collected in Kazakhstan identified anomocytic stomata, simple and stellate trichomes, calcium oxalate druses, and characteristic anatomical features of leaves, stems, sepals, and roots [42]. Histochemical analyses additionally demonstrated the localization of phenolic compounds and flavonoids in petiole and root tissues.

Collectively, these morphological and anatomical observations provide useful criteria for botanical authentication and pharmacognostic standardization of *Abutilon* medicinal plant materials. However, individual microscopic features may occur across multiple related taxa and should therefore be interpreted in combination rather than as universally species-specific diagnostic markers. Comparative anatomical studies supported by authenticated reference specimens and, where appropriate, molecular identification methods would further strengthen species-level authentication and reduce the risk of botanical misidentification.

## 4. Ethnomedical Uses of the Genus *Abutilon*

Species of the genus *Abutilon* have a long history of use in traditional medical systems across Asia, Africa, and other regions. Different plant parts, including leaves, roots, seeds, flowers, and bark, have been prepared as decoctions, infusions, powders, and pastes for a variety of traditional therapeutic purposes [16]. For example, *A. fruticosum* has been documented in an ethnobotanical survey conducted in Hormozgan Province, Iran, where its flowers, leaves, and seeds were reported in traditional preparations for wounds, acne, pustules, colds, and bronchitis, as well as for emollient purposes [43]. These applications represent documented traditional uses rather than evidence of clinically established therapeutic efficacy.

Among the more extensively documented species, *A. theophrasti* has a long history of traditional medicinal use, particularly in Chinese medicine and regional folk practices [13,44,45,46]. In an ethnobotanical survey conducted in Shexian County, Hebei Province, China, Bai et al. [44] documented the traditional use of *A. theophrasti* seeds prepared as decoctions for dysentery, stranguria, and pyogenic infections. The same study also recorded the consumption of tender leaves as juice or a tea substitute and fruits as raw food, illustrating the broader ethnobotanical significance of this species. Other traditional applications have involved roots, seeds, leaves, and aerial parts. According to ethnomedicinal accounts summarized by Li et al. [13], the roots have been used for urinary complaints, including difficult or painful urination, whereas the seeds have been associated with traditional treatments for dysentery and other urinary or inflammatory conditions. Fresh leaves have also been applied externally, including preparations combined with honey or brown sugar, for carbuncles and related skin conditions. Hassan et al. [45] additionally described traditional applications for chest infections, urethritis, toothache, hemorrhoids, gonorrhea, and hemorrhagic conditions, together with anthelmintic, laxative, demulcent, and diuretic uses. Koli et al. [46] reported the use of aerial parts as expectorant and emollient remedies and described applications for rheumatic pain, arthrosis, bruises, sprains, dysentery, otitis media, tinnitus, and hearing-related complaints. These reports document traditional applications rather than clinically established therapeutic effects, and the available information does not support attributing every preparation or indication to a single geographical or medical tradition.

*A. indicum* (L.) Sweet, commonly known as “Atibala,” is widely recognized in Ayurvedic medicine. Its leaves, flowers, seeds, roots, and bark have been traditionally used in preparations intended for inflammatory conditions, pain, wounds, gastrointestinal complaints, and other disorders [12,47,48,49]. The diversity of plant parts and preparations highlights the importance of botanical authentication and preparation-specific chemical characterization when evaluating the pharmacological relevance of these uses.

In Europe and North America, *A. theophrasti* is widely recognized as an agricultural weed [50]. Archaeobotanical findings, including carbonized seeds recovered from a Late Neolithic site in southern Hungary, indicate a long history of human association with this species [51]. However, such archaeological evidence does not independently establish medicinal use and should be distinguished from ethnomedical documentation.

In African traditional medicine, *A. mauritianum* has been reported in preparations used for fever, respiratory and gastrointestinal complaints, dysuria, and skin conditions [14]. Traditional applications of *A. hirtum* have also been documented, including the use of roots as expectorant, analgesic, and antipyretic remedies and leaves or flowers for abscesses [32]. *A. pannosum* has been reported in traditional Indian medicine for dysentery, gonorrhea, and related complaints [52].

The principal ethnomedicinal applications of selected *Abutilon* species, including plant parts, traditional preparations, reported uses, and geographical or medical traditions, are summarized in Table 1.

Collectively, these reports demonstrate considerable diversity in the traditional applications of *Abutilon* species. Recurrent uses for inflammatory, gastrointestinal, infectious, respiratory, and skin-related conditions provide an ethnopharmacological rationale for targeted experimental investigation. Nevertheless, similarities in traditional indications do not establish comparable chemical composition, pharmacological potency, or therapeutic efficacy across species. Future ethnopharmacological research should therefore document botanical identity, preparation methods, administered quantities where available, and the cultural and geographical context of use, while distinguishing traditional knowledge from experimentally validated biological effects.

## 5. Pharmaceutical Quality Control and Formulation Development of the Genus *Abutilon*

The pharmaceutical development of *Abutilon*-derived preparations requires reliable botanical authentication, reproducible raw-material quality assessment, validated analytical methods, and appropriate formulation characterization. Although selected species have been investigated for their pharmacognostic characteristics and preliminary formulation feasibility, the available evidence remains fragmented and predominantly exploratory. This section therefore evaluates the contribution of existing studies to pharmaceutical quality control and early-stage formulation development, while distinguishing experimentally established findings from requirements that have not yet been adequately addressed.

Pharmacognostic investigations have provided preliminary reference characteristics for the authentication of different organs of *A. indicum*. In addition to the reported root characteristics [53], Chakraborthy and Ghorpade [54] examined stem material through macroscopic and microscopic evaluation, quantitative measurements, physicochemical assessment, fluorescence analysis, and preliminary phytochemical screening. The stem powder exhibited a total ash content of 13%, an acid-insoluble ash content of 0.33%, and a loss on drying of 11.37%. Microscopic observations further documented covering trichomes, vascular elements, fibres, and calcium oxalate crystals. These findings provide organ-specific reference parameters for botanical raw-material identification. However, their reproducibility across independently collected samples and their ability to discriminate *A. indicum* from related or substitute species remain insufficiently established.

More recent analytical investigations have extended quality-control approaches beyond morphological identification. Rahate et al. [55] developed an HPTLC–UV densitometric method for six specialized metabolites—20-hydroxyecdysone, vasicine, vasicinone, cryptolepine, quindolinone, and cryptolepinone and applied it to four *Sida* species and *A. indicum*. Among the six investigated analytes, only cryptolepine was reported above the detection threshold in the analyzed *A. indicum* samples, with concentrations of 0.26–0.36 mg/g in aerial parts and 0.32–0.33 mg/g in roots. Complementary chromatographic profiling and chemometric analyses supported the investigation of chemical differences among botanically related raw materials. However, some reported inter-day precision values were comparatively high, and the method should not be interpreted as establishing universal species authentication or comprehensive chemical standardization. Its principal relevance lies in illustrating how targeted chemical profiling can complement botanical identification and help assess potential substitution or adulteration.

Additional pharmacognostic quality parameters have been established for *A. indicum* fruits. Bolleddu et al. [56] reported diagnostic powder-microscopic characteristics and physicochemical measurements, including loss on drying (7.7%), total ash (10.5%), acid-insoluble ash (2.4%), water-soluble extractives (9.64%), and alcohol-soluble extractives (9.04%). The investigation also documented distinct HPTLC fingerprint profiles for alcoholic and hydroalcoholic fruit extracts. These findings provide preliminary reference data for fruit-specific raw-material assessment. Nevertheless, the reported measurements represent characteristics of the investigated material rather than universally validated pharmacopoeial specifications, and the chromatographic fingerprints were not supported by comprehensive quantitative identification of individual marker compounds.

Beyond analytical characterization, formulation-oriented research has explored the incorporation of *A. indicum* leaf extracts into polymeric materials. Tharakeswari et al. [57] prepared poly(vinyl alcohol)-based nanofibrous mats by electrospinning and evaluated their morphology, infrared spectral characteristics, swelling behavior, thickness, mechanical properties, and antibacterial activity. The 50:50 polymer-to-extract preparation exhibited the highest reported swelling ratio (525%) and an inhibition zone of 23 ± 0.61 mm against *Escherichia coli*. However, increasing extract incorporation did not produce uniformly superior mechanical characteristics, and this study did not establish controlled release of specific phytochemical constituents, quantitative extract standardization, biocompatibility, or in vivo therapeutic efficacy. Consequently, these findings demonstrate preliminary material-formulation feasibility rather than validated pharmaceutical delivery performance.

A more directly validated analytical approach was reported by Hussain et al. [58], who developed an HPTLC method for the simultaneous determination of gallic acid and quercetin in a methanolic whole-plant extract of *A. indicum*. The reported concentrations were 0.69% and 0.57% (*w*/*w*), respectively. The method employed densitometric detection at 270 nm and was evaluated for linearity, precision, accuracy, recovery, robustness, and analytical sensitivity. The reported limits of detection were 23 and 41 ng/band for gallic acid and quercetin, respectively, with corresponding limits of quantification of 69 and 123 ng/band. These results provide a useful basis for targeted chemical marker quantification. Nevertheless, the validation was performed within the investigated analytical conditions, and quantification of two constituents alone does not demonstrate comprehensive phytochemical standardization, batch-to-batch equivalence, or a relationship between marker content and therapeutic efficacy.

Collectively, the available studies illustrate complementary but methodologically distinct stages of pharmaceutical development, ranging from organ-specific botanical authentication and physicochemical assessment to chemical fingerprinting, marker quantification, and exploratory formulation engineering. The strongest analytical contributions concern defined chromatographic measurements, whereas the available formulation evidence remains preliminary. In particular, reproducible extract composition, formulation stability, constituent-release behavior, biocompatibility, and manufacturing consistency have not been sufficiently established across the investigated preparations. These limitations indicate that the existing studies provide a foundation for pharmaceutical quality-control development but do not yet demonstrate pharmaceutical product readiness or clinical therapeutic benefit.

## 6. Phytochemical Composition of the Genus *Abutilon*

Species of the genus *Abutilon* exhibit considerable phytochemical diversity, with numerous secondary metabolites reported across different plant organs and extraction systems [59,60]. The principal constituent classes include flavonoids, phenolic acids, triterpenoids, phytosterols, polysaccharides, and fatty acids. However, the reliability of compound identification varies according to the analytical techniques employed, ranging from preliminary chromatographic screening to structural elucidation using complementary spectroscopic methods.

Flavonoids are among the most extensively investigated secondary metabolites in *Abutilon*. Quercetin, kaempferol, myricetin, luteolin, and their glycosides, including rutin and tiliroside, have been reported in several species, particularly *A. theophrasti* and *A. indicum*. Anthocyanidins such as delphinidin and cyanidin, together with catechin derivatives, have also been described [61,62,63,64,65,66]. Nevertheless, the detection of related flavonoid structures across different studies does not necessarily indicate comparable abundance, as extraction conditions and analytical methods differ substantially.

Histochemical investigation of *A. theophrasti* collected in Kazakhstan additionally demonstrated the localization of flavonoids and phenolic compounds in petiole and root tissues [42]. Although such observations provide useful information on the distribution of phytochemical classes within plant organs, histochemical staining alone does not establish the identities or concentrations of individual metabolites.

Phenolic acids constitute another frequently reported group of constituents. Gallic, caffeic, ferulic, protocatechuic, syringic, vanillic, and *p*-hydroxybenzoic acids have been identified or reported in selected *Abutilon* species [64,67,68,69,70]. However, differences in chromatographic resolution, reference-standard availability, and structural confirmation limit direct comparisons of individual phenolic profiles among investigations.

Triterpenoids and phytosterols are also recurrent constituents. Oleanolic acid, lupeol, lupenone, β-sitosterol, stigmasterol, and related sterol glycosides have been reported in *A. theophrasti* and *A. indicum* [71,72,73,74,75,76]. Studies combining chromatographic isolation with NMR and mass spectrometric characterization provide stronger structural evidence than those relying primarily on retention behavior or preliminary chromatographic comparisons.

Fatty acids and related lipid constituents have been characterized in *A. theophrasti* [71,77]. Phytochemical profiling of *A. figarianum* by Saleem et al. [78] additionally revealed solvent-dependent differences in the composition of methanolic and dichloromethane whole-plant extracts. The methanolic extract exhibited higher total phenolic (51.92 ± 0.80 mg GAE/g extract) and flavonoid contents (72.59 ± 0.47 mg QE/g extract) than the dichloromethane extract (18.71 ± 0.43 mg GAE/g and 40.43 ± 0.32 mg QE/g extract, respectively). UHPLC-MS profiling yielded tentative annotations of 11 metabolites in the methanolic extract and 12 in the dichloromethane extract, including flavonoid, alkaloid, coumarin, phenolic, and fatty-acid derivatives. Because these assignments were based primarily on database-assisted mass-spectrometric matching, they should not be interpreted as unequivocally confirmed molecular structures.

A water-soluble galactomannan has also been isolated from *A. indicum* seeds [79], while unusual fatty acids have been reported in its seed oil [80]. These findings indicate that the chemical diversity of *Abutilon* extends beyond conventional phenolic and terpenoid secondary metabolites to include structurally distinct lipid and polysaccharide constituents.

Beyond the major phenolic, terpenoid, and lipid classes, targeted metabolomic analysis has also provided evidence for the occurrence of the indoloquinoline alkaloid cryptolepine in *A. indicum*. Rahate et al. [55] investigated six selected metabolites using HPTLC-UV densitometry and complementary chromatographic and mass-spectrometric analyses. Cryptolepine was the only targeted analyte quantified above the reported detection threshold in the examined *A. indicum* samples, with concentrations of 0.26–0.36 mg/g in aerial parts and 0.32–0.33 mg/g in roots. However, these results were obtained from a limited number of geographically sourced samples, and further independent investigations are required to establish the consistency of cryptolepine occurrence across *A. indicum* populations.

A range of analytical approaches, including GC-MS, HPLC, LC-MS/MS, and NMR spectroscopy, has been employed to investigate the phytochemical composition of *Abutilon* species [63,64,66,72]. However, these techniques provide different levels of structural certainty. Chromatographic and mass spectrometric profiling can support compound annotation, particularly when reference standards and diagnostic fragmentation data are available, whereas comprehensive structural elucidation of isolated constituents generally requires complementary spectroscopic evidence. Accordingly, compounds identified tentatively by untargeted profiling should be distinguished from those confirmed using authentic standards or detailed spectroscopic characterization.

The major phytochemical constituents reported in selected *Abutilon* species, together with their analytical methods and extraction systems, are summarized in Table 2.

The phytochemical evidence summarized in Table 2 demonstrates considerable variation in analytical rigor and chemical standardization. Studies involving chromatographic isolation followed by NMR and mass spectrometric analysis generally provide stronger structural support than investigations relying exclusively on GC-MS library matching or LC-MS-based tentative annotation. Nevertheless, the use of multiple analytical techniques does not automatically establish unequivocal compound identity, particularly when authentic reference standards, stereochemical characterization, or orthogonal confirmation are unavailable.

Quantitative chemical characterization also remains uneven across the available literature. For example, rutin was quantified at approximately 0.38% in a standardized aqueous extract of *A. theophrasti* [72], while quercetin was reported at 3.65% (*w*/*w*) in an ethanolic extract of *A. indicum* [85]. These values demonstrate the feasibility of marker-based characterization but should not be interpreted as directly comparable measures of phytochemical abundance because the investigated species, plant materials, extraction procedures, and analytical methods differed. Furthermore, quantification of one or a few marker compounds does not establish comprehensive chemical standardization of a complex botanical preparation.

An additional limitation concerns the interpretation of phytochemical diversity in relation to pharmacological activity. Although several reported constituents possess independently documented biological properties, their detection in *Abutilon* extracts does not demonstrate that they are responsible for the activities observed in crude preparations. Establishing such relationships requires bioactivity-guided fractionation, quantitative constituent analysis, direct testing of purified compounds, and assessment of potential interactions among metabolites.

Overall, the available literature supports substantial phytochemical diversity across the genus *Abutilon*, but differences in botanical material, extraction procedures, analytical identification criteria, and quantitative reporting limit cross-study comparability. Future investigations should prioritize authenticated plant materials, validated chromatographic methods, orthogonal structural confirmation, quantitative chemical fingerprinting, and batch-to-batch standardization. These approaches are essential for establishing reproducible phytochemical profiles and strengthening the connection between chemical composition and experimentally observed pharmacological activity.

## 7. Pharmacology and Toxicology of the Genus *Abutilon*

A broad range of pharmacological activities has been reported for *Abutilon* species in in vitro and in vivo experimental studies. These include antioxidant, anti-inflammatory, antimicrobial, hepatoprotective, gastroprotective, antidiabetic, cytotoxic and antitumor, neuropharmacological, analgesic, anticonvulsant, and respiratory-related activities [96,97]. The reported effects have been investigated mainly using crude extracts and fractions containing diverse phytochemical constituents, including flavonoids, phenolic compounds, triterpenoids, phytosterols, polysaccharides, and fatty acids. However, the contribution of individual constituents to many of these activities remains incompletely characterized.

Recent research has also extended to plant-mediated nanoparticles and other derived systems, broadening the investigation of *Abutilon*-based materials beyond conventional extracts [97]. Toxicological studies have simultaneously highlighted the importance of dose and preparation type when evaluating the biological effects and safety of *Abutilon*-derived materials [98].

**Figure 3:**
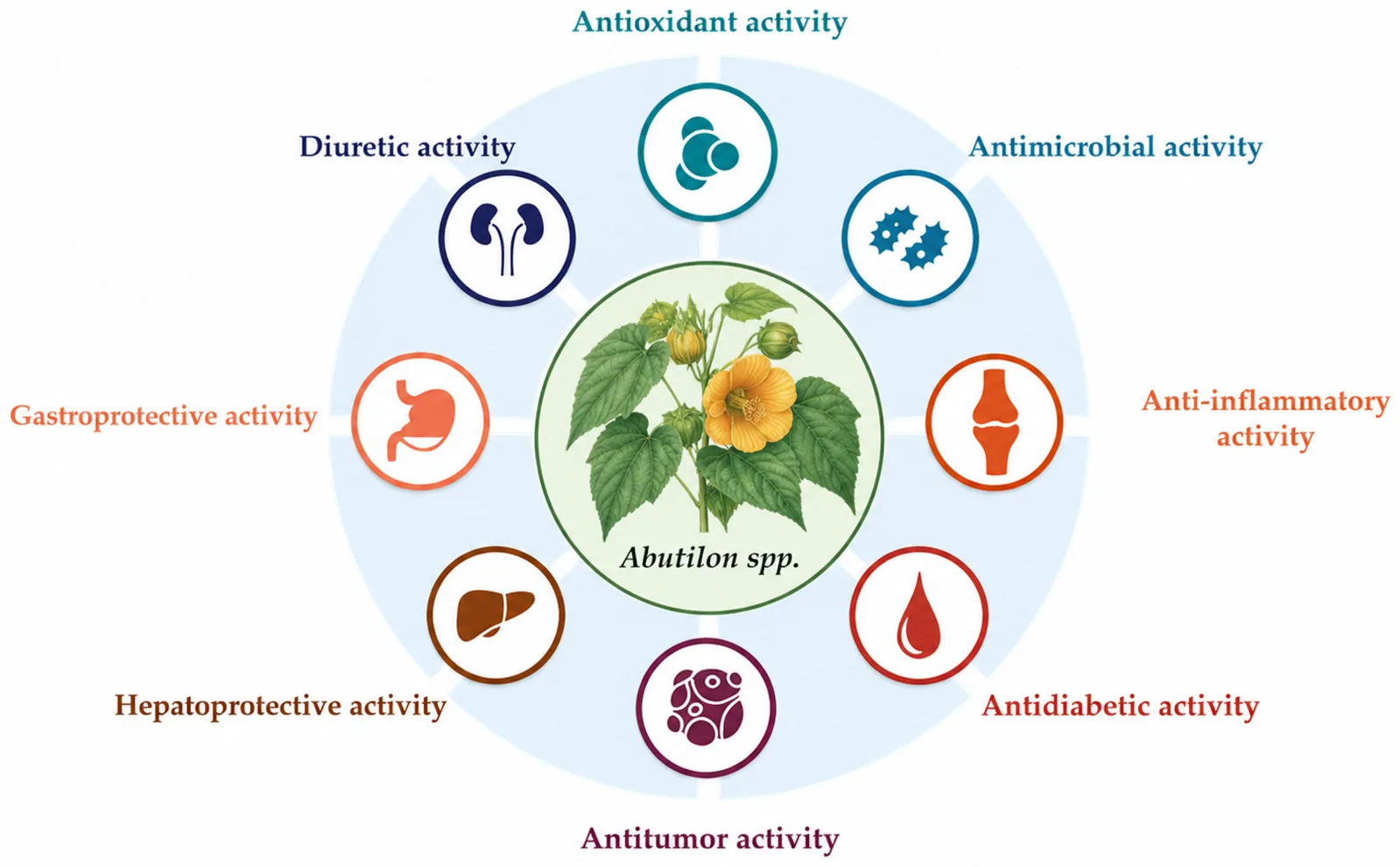
Major pharmacological activities reported for species of the genus *Abutilon*.

The major pharmacological activities reported for species of the genus are illustrated in Figure 3. The following subsections critically examine the available pharmacological and toxicological evidence, with particular emphasis on quantitative outcomes, experimental models, mechanistic interpretation, methodological limitations, and preparation-specific safety concerns. Table 3 provides a critical comparative summary of selected pharmacological studies, highlighting principal outcomes, experimental models, and methodological limitations. Additional pharmacological investigations are presented in Supplementary Table S1, while Section 7.18 critically evaluates the available toxicological evidence.

### 7.1. Antioxidant Activity

Among *Abutilon* species, *A. indicum* has been the most extensively investigated for antioxidant activity. A methanolic leaf extract exhibited 49.62% superoxide radical scavenging and 28.74% nitric oxide radical scavenging at 250 μg/mL [99]. A crude saponin-enriched leaf extract demonstrated concentration-dependent DPPH radical-scavenging activity, increasing from 15.7% at 0.25 mg/mL to 96.17% at 2.5 mg/mL [100]. Phenolic extracts obtained from leaf-derived callus cultures were evaluated using DPPH, total antioxidant capacity (TAC), and reducing-power assays, with DPPH radical inhibition reaching approximately 60–70% [101]. Additional studies employing DPPH, ABTS, superoxide, nitric oxide, hydrogen peroxide, and FRAP assays reported antioxidant activity in *A. indicum* leaf extracts [102,103,104,105]. One investigation reported a DPPH IC_50_ of 89.49 ± 1.23 μg/mL [102]; however, inconsistencies in the reported concentration–response data and IC_50_ values limit the reliability of this estimate. In another study, ABTS radical-scavenging activity ranged from 34.46% to 71.38% across the tested concentrations [103].

Antioxidant activity has also been examined in other plant organs and preparations of *A. indicum*. Methanolic, hydroalcoholic, and aqueous stem extracts were assessed for total phenolic content using the Folin–Ciocalteu method and radical-scavenging activity using the DPPH assay, with the aqueous extract showing the highest reported values among the tested preparations [106]. Hydroethanolic fruit and leaf extracts were investigated using DPPH and FRAP assays. At 50 μg/mL, DPPH radical inhibition was 57.97% for the fruit extract and 37.8% for the leaf extract [107]. An ethanolic flower extract also demonstrated antioxidant activity in DPPH and ABTS assays [108]. In a Wistar rat model of CCl_4_-induced oxidative stress, administration of an ethanolic extract at 500 mg/kg increased hepatic glutathione levels and antioxidant enzyme activities, including superoxide dismutase, catalase, and peroxidase [109]. Additional radical-scavenging effects were reported for different solvent fractions using ABTS, DPPH, and FRAP assays [110].

In *A. theophrasti*, phenolic acids isolated from methanolic leaf extracts, including gallic, protocatechuic, syringic, and vanillic acids, exhibited radical-scavenging and reducing activities in DPPH, ABTS, and FRAP assays [68]. A proanthocyanidin-rich leaf fraction also demonstrated antioxidant activity in ABTS and DPPH assays [82]. Furthermore, aqueous, methanolic, and ethanolic leaf extracts showed concentration-dependent radical-scavenging activity, with reported inhibition values ranging from 62.37% to 81.86% over the tested concentration range of 0.06–1.89 mg/mL [111].

Antioxidant activity has additionally been reported for several less extensively investigated species. Extracts of *A. bidentatum* exhibited antioxidant activity in vitro [112]. In *A. figarianum*, Saleem et al. [78] compared methanolic and dichloromethane whole-plant extracts using six complementary in vitro antioxidant assays. The methanolic extract exhibited substantially higher DPPH (66.64 ± 1.42 mg TE/g extract), ABTS (120.92 ± 1.99 mg TE/g extract), FRAP (123.16 ± 5.73 mg TE/g extract), and CUPRAC (204.26 ± 0.34 mg TE/g extract) responses than the dichloromethane extract. Conversely, the dichloromethane extract showed greater ferrous ion-chelating activity (51.57 ± 0.25 versus 40.15 ± 0.34 mg EDTAE/g extract). These findings demonstrate solvent- and assay-dependent differences in antioxidant capacity; however, they are limited to cell-free biochemical systems and do not establish intracellular antioxidant protection or therapeutic efficacy. In *A. pannosum*, methanolic and ethanolic extracts and solvent-derived fractions demonstrated radical-scavenging activity, with an ethyl acetate fraction producing 86.3% DPPH inhibition in one comparative investigation [113,114,115]. Ethanolic, aqueous, and ethyl acetate leaf extracts of *A. hirtum* also exhibited antioxidant activity in DPPH, superoxide, and FRAP assays [8,116]. Root-derived fractions of *A. muticum* (currently treated as a synonym of *A. pannosum* var. *pannosum*) showed antioxidant activity, while its seed oil was evaluated using ABTS, FRAP, and DPPH assays [110,117]. Dose-dependent radical-scavenging activity was also reported for *Herissantia crispa* (L.) Brizicky, described as *A. crispum* in the original study [118].

Collectively, these investigations indicate antioxidant potential across several *Abutilon* species, although the biological relevance and methodological strength of the available evidence vary considerably among experimental models. Most findings are derived from chemical assays, particularly DPPH, ABTS, and FRAP, which assess radical-scavenging or reducing capacity rather than antioxidant efficacy in biological systems. Furthermore, many crude extracts and enriched fractions remain insufficiently characterized, preventing attribution of the observed effects to specific constituents. The inconsistent IC_50_ reporting in one *A. indicum* investigation [102] further limits quantitative interpretation. In contrast, the restoration of hepatic antioxidant defenses in CCl_4_-exposed rats [109] provides additional biological evidence, although it does not establish direct molecular target engagement or clinical efficacy. Differences in plant organs, extraction procedures, concentrations, and assay conditions also preclude reliable cross-study potency comparisons. The contrasting radical-scavenging and metal-chelating responses observed for *A. figarianum* [78] further illustrate that antioxidant rankings may differ across assay mechanisms, even when the same plant material is investigated.

Future investigations should prioritize chemically standardized preparations, concentration–response characterization, complementary cellular and in vivo models, and pharmacokinetic assessment. Mechanistic studies should distinguish direct radical-scavenging effects from modulation of endogenous antioxidant defense systems and determine whether changes in antioxidant enzyme activities are causally involved in the observed biological responses. Overall, the available evidence supports preliminary antioxidant potential of selected *Abutilon* preparations but remains insufficient to establish therapeutic antioxidant efficacy in humans.

### 7.2. Anti-Inflammatory Activity

The anti-inflammatory activity of *Abutilon* species has been investigated using biochemical screening assays, experimental models of inflammation, and molecular inflammatory markers. Most studies have focused on *A. indicum*, while additional evidence has been reported for *A. theophrasti* and *A. hirtum*. These investigations provide complementary but methodologically heterogeneous evidence of anti-inflammatory activity.

In *A. indicum*, an ethanolic leaf extract administered orally at 300 mg/kg reduced carrageenan-induced paw edema by 64.04% in rats [119]. Additional in vitro investigations evaluated protein denaturation, erythrocyte membrane stabilization, and enzyme inhibition. An acetone leaf extract inhibited albumin denaturation by 58.51–71.28% at concentrations of 2.5–10 µg/mL [120], while another preparation exhibited dose-dependent inhibition of bovine serum albumin denaturation, with a reported IC_50_ of 227 µg/mL [121]. Chloroform, ethanolic, and aqueous leaf extracts inhibited hypotonicity-induced erythrocyte hemolysis by 57.25 ± 0.2%, 55.86 ± 0.2%, and 42.52 ± 0.4%, respectively [122]. An ethanolic leaf extract also inhibited 5-lipoxygenase (5-LOX), with an IC_50_ of 8.89 µg/mL compared with 8.14 µg/mL for curcumin under the same assay conditions [123]. However, protein-denaturation and membrane-stabilization assays are indirect biochemical screening methods and do not independently establish suppression of inflammatory processes in vivo.

Additional evidence has been obtained from inflammation-associated molecular measurements. In an isoniazid-induced experimental model, an ethanolic leaf extract of *A. indicum* administered at 250 and 500 mg/kg reduced TNF-α and NF-κB gene expression and increased IκB expression, while changes in TGF-β were less consistent [124]. These findings suggest modulation of inflammatory signaling under chemically induced injury conditions, although changes in gene expression do not establish direct inhibition of NF-κB signaling proteins or demonstrate a causal mechanism.

Anti-inflammatory activity has also been investigated for isolated constituents of *A. indicum*. Wahab et al. [74] evaluated AB-01, identified as 5′-hydroxyhexyl *n*-hexadecanoate (an aliphatic ester), and AB-05, identified as *n*-octanoyl-β-D-glucopyranosyl-(2′→1″)-β-D-glucopyranosyl-(2″→1‴)-β-D-glucopyranosyl-(2‴→1⁗)-β-D-glucopyranoside (an acylated tetraglucoside). Both compounds reduced carrageenan-induced paw edema in experimental animals at the tested doses of 5 and 10 mg/kg. Although their structures were characterized using spectroscopic methods, the exact purity of the isolated compounds was not established. Molecular docking predicted possible interactions with COX-2 and TNF-α-associated targets; however, these computational findings were not supported by direct target-binding or enzyme-inhibition experiments. Consequently, the molecular mechanisms responsible for the observed anti-inflammatory responses remain unresolved.

In *A. theophrasti*, a total flavonoid fraction obtained from leaves was evaluated in a lipopolysaccharide (LPS)-induced acute lung injury model in mice at doses of 0.25, 0.5, and 1.0 g/kg [125]. Treatment attenuated inflammatory injury and reduced inflammatory cytokine levels, together with iNOS and COX-2 expression and NF-κB/MAPK-associated signaling responses. The combination of animal-level outcomes and molecular inflammatory markers provides comparatively stronger mechanistic evidence than biochemical screening assays alone. Nevertheless, the direct molecular targets and the contributions of individual flavonoid constituents were not established.

In *A. hirtum*, a total ethanolic leaf extract and its chloroform fraction were evaluated in a carrageenan-induced paw edema model at 300 mg/kg [126]. Both preparations produced 50.82% edema inhibition after 5 h. Although the reported effect approached that of the reference anti-inflammatory treatment within the same study, inconsistencies in the reporting of the reference drug limit comparative interpretation. Moreover, these results do not establish pharmacological equivalence to conventional anti-inflammatory agents.

Collectively, the available studies support preliminary anti-inflammatory activity of selected *Abutilon* preparations, with evidence ranging from biochemical screening to animal experiments incorporating molecular inflammatory markers. The 5-LOX inhibition reported for *A. indicum* [123] provides enzyme-level evidence, whereas the changes in TNF-α, NF-κB, IκB, iNOS, COX-2, and MAPK-associated responses [124,125] offer additional mechanistic context. However, these observations do not establish a unified molecular mechanism, and differences in extract composition, experimental models, dosing regimens, and reference controls prevent reliable cross-study potency comparisons.

Future investigations should prioritize chemically standardized extracts, concentration–response characterization, direct target-engagement studies, validation of signaling pathways using selective inhibitors or genetic approaches, pharmacokinetic assessment, and repeated-dose safety evaluation. Overall, the available evidence remains preclinical and is insufficient to establish clinical anti-inflammatory efficacy or therapeutic equivalence to established treatments.

### 7.3. Antimicrobial Activity

Antimicrobial activity has been investigated in several *Abutilon* species, primarily through in vitro susceptibility assays involving crude extracts, solvent-derived fractions, and isolated phytochemical constituents. Although inhibitory effects have been reported against Gram-positive and Gram-negative bacteria and selected fungi, substantial differences in extract composition, microbial strains, and susceptibility-testing methods limit direct comparisons among studies.

In *A. indicum*, an ethanolic leaf extract exhibited concentration-dependent inhibitory activity against periodontal pathogens, including *Aggregatibacter actinomycetemcomitans*, *Porphyromonas gingivalis*, *Prevotella intermedia*, *Fusobacterium nucleatum*, and *Tannerella forsythia* [127]. Antibacterial activity was also reported for flower extracts against *Escherichia coli* and *Staphylococcus aureus* [128]. Among phenolic compounds isolated from methanolic leaf extracts, caffeic acid exhibited antibacterial activity against *B. subtilis* and *S. aureus*, whereas other phenolic acids showed comparatively weaker effects [69]. Additional investigations described antimicrobial activity of leaf extracts against bacterial and fungal species, with outcomes varying according to extraction solvents and experimental conditions [129,130,131,132].

A crude saponin-enriched leaf fraction of *A. indicum* produced inhibition zones of 17 and 15 mm against *S. aureus* and *E. coli*, respectively, with a reported minimum inhibitory concentration (MIC) of 1.11 mg/mL [100]. However, the fraction was not comprehensively characterized, preventing attribution of the observed activity to individual saponins. Antibacterial activity has additionally been reported for *A. indicum* seed oil against selected Gram-positive and Gram-negative bacteria [117]. A formulation-oriented investigation by Tharakeswari et al. [57] evaluated the antibacterial activity of electrospun poly(vinyl alcohol) nanofibrous mats incorporating *A. indicum* leaf extract. In an agar diffusion assay against *E. coli*, inhibition zones increased from 10 ± 0.06 mm for the 90:10 polymer-to-extract formulation to 23 ± 0.61 mm for the 50:50 formulation, whereas unloaded poly(vinyl alcohol) mats produced no inhibition zone. Nevertheless, these findings reflect the antibacterial response of formulated materials rather than isolated phytochemical constituents. The absence of MIC determination, controlled-release characterization, and relevant infection-model validation limits conclusions regarding pharmaceutical antimicrobial efficacy.

In *A. theophrasti*, a methanolic stem extract produced inhibition zones of up to 18 mm against *S. aureus* and *Klebsiella pneumoniae* [133]. A phenolic leaf extract inhibited *E. coli* and *S. aureus* strains, with MIC values ranging from 1.21 to 4.93 mg/mL [134]. More chemically defined evidence was obtained for hibicuslide C, an isolated constituent that inhibited *Pseudomonas aeruginosa* ATCC 27853 and six multidrug-resistant clinical isolates, with MIC values of 5–10 µg/mL [135]. This study also reported bacterial DNA fragmentation, reduced preformed biofilm biomass, and enhanced antibacterial effects in selected antibiotic combinations. Although these findings provide additional mechanistic information, the primary molecular target of hibicuslide C and its efficacy in infection models remain unconfirmed. Antimicrobial effects have also been reported for seed-derived preparations of *A. theophrasti* [136].

Antimicrobial activity has also been investigated in *A. fruticosum*. Mushtaq et al. [137] evaluated extracts from different plant organs using polar and non-polar solvents against four bacterial and two fungal strains. A chloroform root extract produced an inhibition zone of 47 ± 0.14 mm against *S. aureus*, while n-hexane root and stem extracts showed inhibition zones of 40 ± 0.11 and 38 ± 0.28 mm, respectively, against *E. coli*. Ethanolic extracts were reported to exhibit MIC values of 1.25 mg/mL against bacterial strains and 2.5 mg/mL against fungal strains, while the ethanolic fruit extract showed growth inhibition at 0.625 mg/mL against selected bacterial strains. However, MIC estimation was based on an agar diffusion approach, and the reported values require confirmation using standardized dilution-based susceptibility methods.

An ethyl acetate extract of the aerial parts of *A. fruticosum* inhibited selected Gram-positive and Gram-negative bacteria, with MIC values ranging from 0.39 to 12.5 mg/mL. The lowest MIC for the unfractionated extract was observed against *B. subtilis* (0.39 mg/mL). Following fractionation, a 60% ethyl acetate fraction exhibited activity against *Streptococcus pneumoniae*, with an MIC of 0.39 mg/mL and an inhibition zone of 20 mm [138]. Gouda et al. [139] additionally investigated the phytochemical profile and antimicrobial properties of *A. fruticosum* collected from Gebel Elba, Egypt. Nevertheless, the chemical constituents responsible for the observed effects and their biological selectivity remain insufficiently established.

Antibacterial activity has also been investigated in *A. pannosum*. Aadesariya et al. [140] evaluated aqueous, methanolic, petroleum ether, and isopropanolic leaf extracts using agar-well diffusion assays against 11 bacterial strains. Inhibition zones of approximately 9–14 mm were reported against selected susceptible bacteria, including *Enterococcus faecalis*, *Staphylococcus epidermidis*, *Salmonella Typhi*, *Streptococcus pneumoniae*, and *Pseudomonas aeruginosa*. Although the experimental tables reported concentrations of 10–250 µg/mL, the abstract described an initial extract concentration of 1 mg/mL, indicating inconsistent concentration reporting. MIC values were not determined, limiting quantitative interpretation of antibacterial potency.

A methanolic leaf extract of *A. pannosum* exhibited antibacterial activity against eight Gram-positive and Gram-negative bacterial species in an agar-well diffusion assay, producing inhibition zones of 17.1–23.5 mm at a reported extract concentration of 25 mg/mL [141].

In *A. hirtum*, a crude extract and chromatographic fraction AF9 inhibited multidrug-resistant *Salmonella* isolates, with MIC values of 150–300 µg/mL and minimum bactericidal concentrations (MBCs) of 300–600 µg/mL [142].

More recent investigations of *A. pannosum* reported that the antibacterial effects of 70% ethanolic leaf extracts varied according to collection season and bacterial isolate [143]. At subinhibitory concentrations, these preparations enhanced bacteriophage-mediated suppression of *E. coli* O157, although definitive synergistic interactions were not established.

In *A. bidentatum*, solvent-derived leaf extracts exhibited antibacterial activity against selected Gram-positive and Gram-negative bacteria in agar-well diffusion assays. The ethanolic extract was reported to exhibit significant antibacterial activity, with penicillin potassium and streptomycin sulphate serving as reference antibiotics [144]. However, the available information does not provide sufficiently verified quantitative data for reliable extract-specific potency comparisons. A more chemically defined investigation identified abubidentin A, an oleanane-type triterpene ester isolated from the aerial parts of *A. bidentatum*, which inhibited *E. coli*, *P. aeruginosa*, and *S. aureus*, with reported MIC values of ≤150, ≤125, and ≤125 µg/mL, respectively [34]. Antifungal activity against *Fusarium moniliforme* was also reported for an ethanolic extract of the aerial parts of *A. bidentatum* [145]. However, identical inhibition-zone values were presented in another publication by the same author [91], indicating overlapping experimental data that should not be treated as independent evidence.

Collectively, these investigations provide preliminary evidence of antimicrobial activity across several *Abutilon* species, but the strength and pharmacological relevance of the findings vary substantially. Most studies rely on in vitro assays using crude or incompletely characterized extracts. Inhibition-zone diameters obtained from agar diffusion assays cannot be directly compared with MIC or MBC values, and susceptibility breakpoints established for conventional antibiotics should not be applied to botanical preparations.

Studies involving structurally characterized constituents, particularly hibicuslide C [135] and abubidentin A [34], provide more compound-specific evidence than crude-extract screening. However, differences in microbial strains and experimental conditions prevent reliable direct comparisons of their potency. Furthermore, the DNA fragmentation and antibiofilm effects reported for hibicuslide C do not establish its primary molecular target, and enhanced activity in antibiotic combinations requires appropriate interaction analyses before pharmacological synergy can be concluded.

Future investigations should prioritize authenticated botanical materials, chemically standardized preparations, harmonized antimicrobial susceptibility testing, appropriate reference antibiotics, mammalian-cell selectivity assessment, direct molecular target validation, and efficacy studies in relevant infection models. Overall, the available evidence supports preliminary antimicrobial potential of selected *Abutilon* preparations but remains insufficient to establish clinical antimicrobial efficacy.

### 7.4. Hepatoprotective Activity

Hepatoprotective activity has been investigated in several *Abutilon* species, particularly *A. indicum*, using experimental models of chemically induced liver injury. The available studies primarily involve carbon tetrachloride (CCl_4_)-, paracetamol-, and lead acetate-induced hepatotoxicity in animals, with outcomes assessed through serum biochemical markers, oxidative stress parameters, and, in selected investigations, histopathological examination.

In *A. indicum*, an aqueous leaf extract administered orally at 100 and 200 mg/kg attenuated CCl_4_- and paracetamol-induced liver injury in Wistar rats, as indicated by reductions in serum aspartate aminotransferase (AST/SGOT), alanine aminotransferase (ALT/SGPT), alkaline phosphatase (ALP), and bilirubin, together with partial restoration of hepatic glutathione levels [146]. A 70% ethanolic flower extract administered at 100, 250, and 500 mg/kg similarly reduced serum liver injury markers and bilirubin in a CCl_4_-induced hepatotoxicity model [147]. In a separate study, an aqueous root extract administered at 300 and 500 mg/kg attenuated lead acetate-induced hepatic oxidative stress, as reflected by reduced malondialdehyde (MDA) levels and increased superoxide dismutase (SOD), catalase (CAT), and glutathione peroxidase (GPx) activities [148]. Although these findings suggest a possible contribution of antioxidant-associated processes, the active constituents and specific molecular mechanisms remain insufficiently characterized.

Formulation-based approaches have also been investigated. A phytosomal formulation containing combined extracts of *A. indicum* and *Piper longum* was evaluated at 100 mg/kg in a CCl_4_-induced hepatotoxicity model [85]. The formulation improved biochemical markers of hepatic injury and attenuated histopathological alterations, with greater reported effects at a lower administered dose than the corresponding combined extract evaluated at 100, 200, and 400 mg/kg. However, these observations do not independently demonstrate enhanced bioavailability because direct pharmacokinetic measurements were not performed. Furthermore, the formulation contained two botanical species, preventing attribution of the observed hepatoprotective effects specifically to *A. indicum*.

A tablet formulation containing a hydroalcoholic stem extract of *A. indicum* was also evaluated in a paracetamol-induced hepatotoxicity model and was associated with improvements in biochemical markers of liver injury [149]. However, the original publication reported inconsistent administered doses (200 mg/kg in the abstract versus 250 mg/kg in the discussion and conclusion), limiting reproducibility and quantitative interpretation. Consequently, the reported activity should be considered preliminary until the dosing regimen and formulation composition are independently clarified.

Hepatoprotective activity has additionally been reported for other *Abutilon* species. An aqueous leaf decoction of *A. hirtum*, administered at 100 and 200 mg/kg, reduced serum transaminases, ALP, and total bilirubin in a CCl_4_-induced liver injury model, with greater histological improvement reported at the higher dose [150]. Similarly, an 80% aqueous-methanolic extract of the aerial parts of *A. bidentatum*, administered orally at 150, 300, and 500 mg/kg, reduced biochemical indicators of CCl_4_- and paracetamol-induced hepatotoxicity in rabbits [151]. The reported effects included dose-associated reductions in SGOT, SGPT, ALP, and direct bilirubin. Although qualitative histopathological observations supported attenuation of hepatic tissue injury, only one selected animal per group was examined histologically, substantially limiting the strength of the tissue-level evidence.

Collectively, these studies provide preliminary in vivo evidence of hepatoprotective activity in selected *Abutilon* preparations. Concordant improvements in serum liver injury markers and selected histopathological findings offer greater biological support than isolated biochemical measurements. Nevertheless, substantial heterogeneity in plant organs, extraction solvents, dosing regimens, hepatotoxic agents, and reference controls prevents reliable direct comparison of hepatoprotective potency across studies. Most extracts also lack comprehensive quantitative phytochemical standardization, making it difficult to identify active constituents or establish causal molecular mechanisms. Improvements in oxidative stress-related biomarkers should therefore not be interpreted as direct evidence of specific antioxidant signaling pathways or molecular target engagement.

Future studies should prioritize authenticated botanical materials, chemically standardized preparations, controlled dose–response investigations, and complementary models representing chronic and multifactorial liver injury. Comparative pharmacokinetic and pharmacodynamic studies are particularly important for evaluating phytosomal and tablet formulations, while repeated-dose toxicity and mechanistic validation are required to assess translational potential. Overall, the available evidence supports preliminary hepatoprotective potential of selected *Abutilon* species but remains insufficient to establish clinical efficacy, long-term safety, or therapeutic applicability in human liver diseases.

### 7.5. Antidiabetic Activity

Antidiabetic activity has been investigated in *Abutilon* species using biochemical assays, cellular and ex vivo systems, and experimental animal models. Most studies have focused on *A. indicum*, whereas fewer investigations have examined *A. hirtum*. The reported effects include inhibition of carbohydrate-digesting enzymes, modulation of intestinal glucose absorption, changes in cellular glucose regulation, insulin-related responses, and reductions in blood glucose concentrations. However, these findings represent distinct experimental endpoints and should not be interpreted as equivalent evidence of therapeutic antidiabetic efficacy.

In *A. indicum*, an aqueous whole-plant extract reduced postprandial hyperglycemia in diabetic rats [7]. Complementary investigations involving a butanol fraction demonstrated PPARγ-associated transcriptional activity and adipocyte differentiation in 3T3-L1 cells, together with increased GLUT1 promoter activity. However, these observations do not independently establish enhanced GLUT1 protein expression or transporter-mediated glucose uptake, and activation of Akt/GSK-3β signaling was not demonstrated under the reported conditions [7].

A chloroform fraction of *A. indicum* administered orally at 50 mg/kg for 21 days reduced blood glucose concentrations in streptozotocin-induced diabetic rats from 373.6 ± 4.9 to 195.1 ± 3.7 mg/dL, accompanied by improvements in selected lipid and insulin-related parameters [152]. Nevertheless, the preparation was not sufficiently characterized to identify the specific constituents responsible for the observed effects.

Enzyme-inhibitory activity has also been investigated in *A. indicum*. Pant et al. [153] evaluated a methanolic leaf extract using in vitro α-amylase and α-glucosidase inhibition assays. The authors reported IC_50_ values of 129.89 and 137.61 µg/mL, respectively, over a tested concentration range of 10–160 µg/mL. However, the maximum observed inhibition remained below 50% within the reported experimental range, raising concerns regarding the reliability of the calculated IC_50_ values. Consequently, these results should be interpreted as preliminary evidence of enzyme-inhibitory activity rather than robust quantitative estimates of inhibitory potency.

Enzyme-inhibitory activity has also been investigated in *A. figarianum*. Saleem et al. [78] reported α-glucosidase inhibition equivalent to 0.60 ± 0.03 and 0.65 ± 0.02 mmol ACAE/g extract for methanolic and dichloromethane extracts, respectively. The corresponding α-amylase inhibitory responses were 1.72 ± 0.05 and 1.88 ± 0.04 mmol ACAE/g extract. Although the dichloromethane extract showed numerically higher values in both assays, the differences between extracts were not statistically significant. Furthermore, these acarbose-equivalent measurements do not provide IC_50_ values or establish pharmacologically achievable inhibitory concentrations. Accordingly, the findings support preliminary biochemical enzyme-inhibitory activity rather than demonstrated antidiabetic efficacy.

A 50:50 ethanol–water leaf extract of *A. indicum* exhibited 60% inhibition of advanced glycation end-product formation at a reported concentration of 25 µg/mL [154]. The authors additionally reported 71% inhibition of fructosamine formation after three days of incubation. However, detailed quantitative data supporting the fructosamine result were not provided, and the described α-amylase, α-glucosidase, and yeast-cell glucose-uptake assays lacked sufficiently detailed numerical outcomes. These findings suggest preliminary antiglycation potential but do not establish improved glycemic control or therapeutic efficacy in diabetes.

An aqueous extract of *A. indicum* was additionally reported to reduce postprandial glucose concentrations in diabetic rats at 0.5 and 1 g/kg. Complementary ex vivo and cellular investigations indicated inhibition of intestinal glucose absorption and stimulation of insulin secretion [155]. Nevertheless, the relative contributions of these processes to systemic glucose lowering remain incompletely established.

Comparative investigations of *A. indicum* leaf extracts have revealed extraction-dependent differences in glucose-lowering responses. In healthy rats, petroleum ether, benzene, alcoholic, and aqueous extracts administered at 400 mg/kg produced reported glucose reductions of 34.69%, 46.34%, 30.30%, and 53.55%, respectively [156]. In another study, ethanolic and aqueous leaf extracts at 400 mg/kg produced significant hypoglycemic effects, whereas less polar preparations showed weaker or nonsignificant activity [157]. Importantly, findings obtained in normoglycemic animals cannot be directly extrapolated to therapeutic efficacy in established diabetes.

In *A. hirtum*, an ethanolic leaf extract and its solvent-derived fractions administered at 300 mg/kg reduced blood glucose levels in streptozotocin-induced diabetic rats, with a crude polysaccharide fraction showing the greatest effect among the investigated preparations [126]. A separate biochemical investigation reported α-amylase inhibition by an *A. hirtum* leaf extract, with an IC_50_ of 119.48 µg/mL compared with 12.53 µg/mL for acarbose [158]. This approximately 9.5-fold difference indicates lower inhibitory potency of the crude extract under the reported assay conditions.

Collectively, the available studies suggest that selected *Abutilon* preparations may influence multiple processes associated with glucose homeostasis. Nevertheless, the evidence varies substantially in biological relevance and methodological reliability. Enzyme-inhibition and antiglycation assays provide preliminary biochemical evidence, whereas animal experiments offer additional functional information. The α-glucosidase and α-amylase inhibition reported for *A. figarianum* [78] provides additional extract-level biochemical evidence, although the acarbose-equivalent responses and absence of experimentally determined IC_50_ values prevent reliable assessment of inhibitory potency or direct comparison with other *Abutilon* preparations. However, reported PPARγ-associated responses, GLUT1 promoter activation, intestinal glucose absorption, and insulin secretion do not establish a unified or causally validated molecular mechanism. In particular, transcriptional reporter activity should not be equated with functional transporter expression, and isolated tissue responses may not reliably predict systemic metabolic effects.

Most investigated preparations remain chemically insufficiently characterized, preventing confident attribution of activity to individual constituents. Furthermore, chemically induced diabetes models do not fully reproduce the heterogeneous pathophysiology of human type 2 diabetes, while inconsistencies in enzyme-inhibition reporting [153] limit quantitative interpretation. Future studies should prioritize chemically standardized preparations, experimentally validated concentration–response relationships, direct molecular target assessment, complementary insulin-resistance models, pharmacokinetic characterization, and repeated-dose safety evaluation. Overall, the available evidence supports preliminary antidiabetic potential of selected *Abutilon* species but remains insufficient to establish clinical efficacy or safety in patients with diabetes mellitus.

### 7.6. Antitumor Activity

Antitumor-related activity of *Abutilon* species has been investigated primarily through in vitro cytotoxicity and antiproliferative assays, with additional evidence from plant-mediated nanomaterials, computational studies, and limited animal experiments. Although several preparations have reduced cancer-cell viability under experimental conditions, these findings do not independently establish tumor selectivity or therapeutic anticancer efficacy.

In *A. indicum*, an ethanolic leaf extract inhibited proliferation of A549 human lung cancer cells, with a reported IC_50_ of 85.2 µg/mL compared with 24.9 µg/mL for cisplatin under the same assay conditions [123]. At 200 µg/mL, the extract produced 72.1% growth inhibition. These findings indicate measurable antiproliferative activity but lower potency than the reference drug under the investigated conditions.

Iron oxide nanoparticles synthesized using an aqueous leaf extract of *A. indicum* exhibited concentration-dependent cytotoxicity against MDA-MB-231 breast cancer cells [159]. In the MTT assay, cell viability decreased from 96% at 10 µg/mL to 13% at 100 µg/mL. Apoptosis-associated morphological changes were additionally observed using acridine orange/ethidium bromide and DAPI staining. However, inconsistencies between the reported IC_50_ values for the nanoparticles and cisplatin, as well as discrepancies in exposure duration (24 versus 48 h), limit reliable quantitative interpretation. Furthermore, the observed effects cannot be attributed exclusively to plant-derived phytochemicals, and tumor selectivity remains unestablished.

Polyphenol-stabilized gold nanoparticles prepared using *A. indicum* leaf extract exhibited cytotoxicity against HT-29 colorectal cancer cells, with IC_50_ values of 210 µg/mL after 24 h and 180 µg/mL after 48 h [160]. The latter study reported increased reactive oxygen species production, disruption of mitochondrial membrane potential, DNA damage, cell-cycle arrest, and activation of apoptosis-associated markers. These observations provide mechanistic context but do not establish that the cytotoxic effects were mediated exclusively by plant-derived constituents. Nanoparticle composition, surface characteristics, and metal-associated toxicity may independently contribute to the observed cellular responses.

Cytotoxic activity has additionally been reported for *A. hirtum*. An ethanolic leaf extract produced 80.46% growth inhibition in MCF-7 breast cancer cells at 4.40 mg/mL after 72 h [8]. Importantly, the same study reported 80.48% growth inhibition in non-malignant human dermal fibroblasts at 2.20 mg/mL, raising concerns regarding cellular selectivity. Because these responses were obtained at different concentrations, they do not permit calculation of a reliable selectivity index; nevertheless, they indicate that substantial cytotoxicity was not restricted to malignant cells. In another investigation, an aqueous extract of *A. hirtum* reduced MCF-7 cell viability, with a reported IC_50_ of 368.7 µg/mL and 43.71% growth inhibition at 300 µg/mL [116].

Computational investigations have explored possible cancer-associated molecular interactions of *A. theophrasti* constituents. In a molecular docking study targeting a protein associated with TGF-β signaling in gastric cancer, myricetin exhibited a predicted binding energy of −8.7 kcal/mol [161]. However, this result represents a computational prediction and does not establish direct target engagement, pathway inhibition, or anticancer activity in biological systems.

In vivo evidence remains limited. An ethanolic extract of the aerial parts of *A. bidentatum* was evaluated in a Sarcoma-180 tumor model at 100 mg/kg/day, with a reported tumor growth ratio of 83.2% [91]. The original investigators classified this result as negative for antitumor activity. This finding is important because it demonstrates that positive cytotoxicity results from screening assays should not be assumed to translate into tumor suppression in vivo.

Collectively, the available evidence is heterogeneous and dominated by preliminary cell-based investigations. Differences in botanical preparations, chemical characterization, cancer cell lines, exposure periods, experimental controls, and assay methodologies prevent reliable direct comparison of IC_50_ values and growth-inhibition percentages across studies. Furthermore, the cytotoxicity observed in non-malignant fibroblasts following exposure to *A. hirtum* extract [8] highlights the importance of distinguishing general cytotoxicity from tumor-selective effects.

Although nanoparticle-based investigations and apoptosis-associated measurements provide additional biological information, they do not establish a unified or causally validated anticancer mechanism. Appropriate controls separating the effects of botanical extracts, inorganic nanomaterials, and combined preparations are necessary. Similarly, molecular docking predictions require experimental confirmation, while the negative animal-model result reported for *A. bidentatum* [91] limits broad claims of antitumor efficacy.

Future investigations should prioritize chemically characterized preparations, isolation and purity assessment of active constituents, comparative cytotoxicity against non-malignant cells, determination of selectivity indices, direct molecular target validation, pharmacokinetic and toxicological evaluation, and efficacy testing in appropriately controlled animal tumor models. Overall, the available findings support preliminary cytotoxic and antiproliferative activity of selected *Abutilon*-derived preparations but remain insufficient to establish selective anticancer efficacy or clinical therapeutic potential.

### 7.7. Neurotropic and Neuroprotective Activities

Neuropharmacological effects have been investigated primarily in *A. indicum*, while preliminary biochemical evidence of cholinesterase inhibition has also been reported for *A. figarianum* [78]. The available evidence is predominantly derived from animal studies assessing behavioral outcomes and selected biochemical markers, with limited characterization of the active constituents and molecular mechanisms responsible for the observed effects.

In an aluminum chloride (AlCl_3_)-induced model of cognitive impairment in Sprague–Dawley rats, a methanolic whole-plant extract of *A. indicum* administered orally at 400 and 600 mg/kg improved selected learning- and memory-related behavioral outcomes [162]. The investigation employed elevated plus maze, radial arm maze, and passive avoidance tests, together with measurement of brain acetylcholinesterase (AChE) activity. Treatment reduced transfer latency and AChE activity relative to the AlCl_3_-exposed control group. However, the behavioral dose–response relationship was not consistent: the 400 mg/kg group exhibited lower transfer latency than the 600 mg/kg group, whereas the higher dose produced a greater reduction in AChE activity. These findings suggest a possible association with cholinergic processes but do not establish a causal mechanism or disease-modifying neuroprotection.

Complementary biochemical evidence has been reported for *A. figarianum*. Saleem et al. [78] evaluated methanolic and dichloromethane whole-plant extracts against acetylcholinesterase (AChE) and butyrylcholinesterase (BChE). The dichloromethane extract exhibited higher inhibitory responses, with AChE and BChE activities of 4.50 ± 0.59 and 4.55 ± 0.26 mg GALAE/g extract, respectively, compared with 3.33 ± 0.08 and 1.67 ± 0.08 mg GALAE/g extract for the methanolic preparation. However, these results were obtained from cell-free biochemical assays and do not establish central nervous system exposure, cholinesterase inhibition in vivo, or neuroprotective efficacy.

Additional neuropharmacological effects were investigated by Zhou et al. [163] using a crude methanolic extract prepared from *A. indicum* leaves and bark. The extract was administered intraperitoneally to Swiss albino mice at 30, 50, and 100 mg/kg. Anxiolytic-like responses were reported in elevated plus maze, light–dark exploration, and hole-board tests, while antidepressant-like responses were observed in forced swimming and tail suspension paradigms. The most pronounced effects were reported at 100 mg/kg. However, these behavioral outcomes do not establish specific anxiolytic or antidepressant molecular mechanisms, and possible effects on general locomotor activity must be considered when interpreting the findings.

Collectively, these investigations provide preliminary in vivo evidence of neuropharmacological activity in *A. indicum* and complementary in vitro biochemical evidence of cholinesterase inhibition in *A. figarianum* [78]. However, improvements in cognitive performance, reductions in brain AChE activity, extract-level cholinesterase inhibition, and anxiolytic- or antidepressant-like behavioral responses represent distinct experimental endpoints and should not be interpreted as equivalent evidence of neuroprotection. In particular, the AlCl_3_-induced cognitive impairment model reproduces selected aspects of neurotoxicity but does not fully reflect the multifactorial pathology of human Alzheimer’s disease. Moreover, biochemical cholinesterase inhibition does not establish central nervous system exposure or neuroprotective efficacy.

The available evidence is further limited by incomplete phytochemical standardization, insufficient direct molecular target validation, and the absence of comprehensive pharmacokinetic and brain-exposure data. Moreover, changes in brain AChE activity do not independently demonstrate direct enzyme inhibition by specific constituents, and behavioral improvements do not establish disease-modifying effects.

Future studies should prioritize chemically standardized preparations, appropriate behavioral and locomotor controls, complementary models of neurological dysfunction, direct assessment of molecular targets, pharmacokinetic and brain-distribution studies, and repeated-dose neurotoxicity evaluation. Overall, the available findings support preliminary neuropharmacological potential of *A. indicum* and biochemical cholinesterase-inhibitory activity of *A. figarianum*, but remain insufficient to establish clinical efficacy or disease-modifying neuroprotective effects.

### 7.8. Anticonvulsant Activity

Anticonvulsant activity of *A. indicum* has been investigated in preclinical seizure models, particularly pentylenetetrazole (PTZ)- and maximal electroshock (MES)-induced seizures in rats. These models assess responses to chemically and electrically induced convulsions, providing complementary experimental evidence of seizure-modifying activity. However, differences in plant organs, extraction procedures, administered doses, and experimental conditions limit direct comparisons among studies.

Mahanthesh and Jalalpure [164] evaluated chloroform, 90% ethanolic, and aqueous stem extracts of *A. indicum* administered orally at 200 mg/kg in Wistar rats. In the MES model, the chloroform extract abolished tonic hind-limb extension, compared with a mean duration of 6.17 s in untreated controls. In the PTZ model, all six rats receiving the chloroform extract survived without observed convulsions, whereas ethanolic and aqueous preparations delayed seizure onset but did not completely prevent convulsions. These findings indicate comparatively greater anticonvulsant activity of the chloroform stem extract under the investigated conditions. However, the evaluation of a single dose and the small experimental groups limit characterization of dose–response relationships and the precision of efficacy estimates.

In a separate study, Golwala et al. [165] investigated ethanolic and aqueous leaf extracts of *A. indicum* administered orally at 100 and 400 mg/kg in PTZ- and MES-induced seizure models. In the PTZ model, the ethanolic extract produced 50% and 67% protection after 24 h at 100 and 400 mg/kg, respectively, compared with 17% and 34% for the corresponding aqueous extract groups. In the MES model, the ethanolic preparation reduced tonic hind-limb extension duration from 15.66 s in controls to 6.17 and 5.17 s at 100 and 400 mg/kg, respectively. These findings suggest dose-associated anticonvulsant effects, with the ethanolic leaf extract generally showing greater activity than the aqueous preparation under the reported conditions.

Collectively, these investigations provide preliminary in vivo evidence of anticonvulsant activity of selected *A. indicum* preparations in chemically and electrically induced seizure models. The chloroform stem extract evaluated by Mahanthesh and Jalalpure [164] prevented observable convulsions in the PTZ model and abolished tonic hind-limb extension in the MES model at 200 mg/kg. In contrast, Golwala et al. [165] reported dose-associated effects of ethanolic and aqueous leaf extracts administered at 100 and 400 mg/kg. However, these findings cannot be directly compared because of differences in plant organs, extraction procedures, experimental protocols, and reference treatments. Furthermore, small experimental groups, inconsistencies in reporting certain experimental parameters, and the absence of direct molecular target validation limit the strength of the evidence. The available results therefore support preliminary anticonvulsant potential but do not establish clinical efficacy or therapeutic equivalence to established antiseizure medications.

Both studies investigated crude extracts with limited quantitative phytochemical characterization. Although chromatographic profiling was performed in the stem-extract study [164], the active constituents responsible for the observed anticonvulsant effects were not experimentally identified. Neither investigation directly established modulation of GABAergic neurotransmission, voltage-gated ion channels, or other specific neuronal targets. Consequently, the observed activity cannot be assigned to a defined molecular mechanism.

Future investigations should prioritize chemically standardized extracts, isolation and testing of active constituents, controlled dose–response experiments, appropriate reference anticonvulsants, direct molecular target validation, and complementary models of recurrent or spontaneous seizures. Pharmacokinetic characterization, brain-exposure assessment, and repeated-dose neurotoxicity evaluation are also necessary to assess translational relevance. Overall, the available evidence supports preliminary anticonvulsant potential of selected *A. indicum* preparations but remains insufficient to establish clinical efficacy, long-term safety, or therapeutic equivalence to established antiseizure medications.

### 7.9. Anti-Asthmatic Activity

The potential anti-asthmatic activity of *A. indicum* has been investigated in a small uncontrolled clinical study and complementary preclinical models. Although these investigations suggest possible respiratory-related effects, their interpretation requires careful consideration of study design, experimental endpoints, and the limited chemical standardization of the investigated preparations.

Paranjape and Mehta [166] conducted an open-label, noncomparative clinical study involving patients aged 15–80 years with mild-to-moderate bronchial asthma. Of 38 enrolled participants, 30 completed the four-week intervention, during which powdered dried aerial parts of *A. indicum* were administered orally at 1 g three times daily. Twenty participants received *A. indicum* alone, whereas ten continued conventional anti-asthmatic medications. Following treatment, mean forced vital capacity (FVC) increased from 1.61 to 1.86 L, and forced expiratory volume in one second (FEV_1_) increased from 1.30 to 1.48 L, with statistically significant within-group changes (*p* < 0.05). Improvements in asthma-related symptoms and maximum voluntary ventilation (MVV) were also reported. However, changes in the FEV_1_/FVC ratio and forced expiratory flow between 25% and 75% of FVC (FEF_25–75%_) were not statistically significant. No adverse effects were reported during the four-week observation period. Nevertheless, the absence of a control group, participant attrition, and concomitant medication use prevent attribution of the observed improvements specifically to *A. indicum* and preclude conclusions regarding long-term safety.

In a complementary experimental investigation, Paranjape and Mehta [167] evaluated a methanolic extract of the aerial parts of *A. indicum* in models of bronchospasm, mast-cell degranulation, and inflammation. Oral administration at 250 and 500 mg/kg did not significantly protect guinea pigs against histamine- or acetylcholine-induced bronchospasm. Similarly, extract concentrations of 250–1000 µg/mL did not significantly inhibit agonist-induced contractions in isolated smooth-muscle preparations. In contrast, the extract reduced compound 48/80-induced and egg albumin-induced degranulation of rat peritoneal mast cells at concentrations of 11.11–33.33 and 10–30 mg/mL, respectively. Oral administration at 250 and 500 mg/kg also attenuated compound 48/80- and carrageenan-induced paw edema, indicating mast-cell-stabilizing and anti-inflammatory activity under the investigated conditions. However, neutrophil adhesion was not significantly altered following administration of 100 or 200 mg/kg/day for eight days.

The clinical and experimental findings provide complementary but methodologically distinct evidence. The clinical study [166] offers direct observations in patients with asthma, but its uncontrolled design and small sample size substantially limit causal interpretation. The experimental study [167] provides functional evidence of mast-cell stabilization and reduced inflammatory responses but does not support a direct bronchodilator effect. Moreover, the relatively high concentrations used in mast-cell assays raise questions regarding pharmacologically achievable exposure, and the reported responses were not linked to experimentally validated molecular targets.

The available studies also investigated different botanical preparations, including powdered aerial parts and a methanolic extract, preventing direct pharmacological comparison. Neither study adequately characterized the active constituents or established the relationship between administered doses, systemic exposure, and respiratory outcomes. Furthermore, acute bronchospasm and general inflammatory models do not fully reproduce the chronic airway inflammation and remodeling associated with human asthma.

Future investigations should prioritize authenticated and chemically standardized preparations, direct assessment of mast-cell-associated molecular pathways, pharmacokinetic and safety studies, and complementary models of chronic allergic airway inflammation. Adequately powered randomized controlled clinical trials with clearly defined background therapies and standardized pulmonary function outcomes are required to determine clinical efficacy. Overall, the available evidence supports preliminary respiratory-related and anti-inflammatory activity of *A. indicum*, but remains insufficient to establish clinically effective anti-asthmatic treatment or equivalence to conventional therapies.

### 7.10. Analgesic Activity

Analgesic activity of *A. indicum* has been investigated in preclinical models of chemically induced and thermal nociception using crude root extracts and isolated phytochemical constituents. These studies provide complementary evidence of antinociceptive activity, although differences in preparation type, experimental endpoints, and dosing limit direct pharmacological comparisons.

Goyal et al. [168] evaluated petroleum ether, methanolic, ethanolic, and aqueous root extracts of *A. indicum* administered orally at 200 and 400 mg/kg in Swiss albino mice. Antinociceptive activity was assessed using acetic acid-induced writhing, tail-flick, and tail-immersion assays. At 400 mg/kg, the petroleum ether extract produced 30.78% inhibition of acetic acid-induced writhing, compared with 25.00% for the aqueous and 14.11% for the ethanolic extracts. Aspirin at 100 mg/kg produced 31.12% inhibition under the same experimental conditions, whereas the methanolic extract did not demonstrate statistically significant activity. The petroleum ether preparation also increased reaction latency in thermal nociception tests, suggesting activity across different nociceptive paradigms. However, the crude extracts were not sufficiently characterized to identify the constituents responsible for the observed effects.

More compound-focused evidence was reported by Wahab et al. [74], who investigated AB-01 and AB-05 isolated from a methanolic extract of *A. indicum*. Both preparations were evaluated at 5 and 10 mg/kg using hot-plate and acetic acid-induced writhing assays. At 10 mg/kg, AB-01 and AB-05 produced reported inhibition values of 71.45% and 74.79%, respectively, in the hot-plate test, compared with 79.21% for tramadol. In the writhing assay, the corresponding reductions were 46.17% and 38.19%, compared with 44.23% for diclofenac under the reported conditions. Although these findings indicate measurable antinociceptive activity, the original study did not establish the exact purity of the isolated constituents, preventing confident attribution of the observed effects exclusively to AB-01 and AB-05.

Collectively, the available studies provide preliminary in vivo evidence of antinociceptive activity in selected *A. indicum* preparations. The use of both chemically induced writhing and thermal nociception assays offers complementary functional information, but these endpoints do not independently establish specific peripheral or central molecular mechanisms. Furthermore, the apparent similarity between selected extract or compound responses and reference drugs within individual experiments should not be interpreted as pharmacological equivalence because the administered doses, chemical composition, and systemic exposure differ.

Although the investigation of AB-01 and AB-05 represents progress toward constituent-level pharmacological assessment, uncertainty regarding compound purity limits mechanistic interpretation. In addition, molecular docking predictions involving inflammation-related targets do not establish direct target engagement or demonstrate a causal contribution to analgesic activity. Differences in botanical preparation, dosing, experimental conditions, and reference treatments also prevent reliable cross-study potency comparisons.

Future investigations should prioritize chemically standardized extracts, independent confirmation of isolated-compound purity, controlled dose–response studies, direct molecular target validation, pharmacokinetic characterization, and repeated-dose safety evaluation. Overall, the available findings support preliminary antinociceptive potential of *A. indicum* root extracts and selected isolated constituents but remain insufficient to establish clinical analgesic efficacy or therapeutic equivalence to established analgesics.

### 7.11. Diuretic Activity

Diuretic activity has been investigated in *A. indicum* and *A. hirtum* using experimental rat models. The available studies assessed urine output, urinary electrolyte excretion, and selected renal biochemical parameters, while additional antiurolithiatic effects were investigated for *A. indicum*. However, differences in botanical preparations, experimental designs, and outcome measurements limit direct comparisons among studies.

In *A. indicum*, an aqueous seed extract administered at 200 and 400 mg/kg increased urine output and urinary sodium excretion in rats [169]. In contrast, no corresponding significant increase in potassium excretion was reported. These findings suggest preliminary diuretic and natriuretic activity, although the specific renal transport mechanisms and consequences for electrolyte balance remain unidentified.

Velraj and Ravichandran [170] evaluated ethanolic and chloroform whole-plant extracts of *A. indicum* at 400 mg/kg in albino rats. Diuretic activity was assessed through urine volume and urinary sodium, potassium, and chloride measurements, whereas antiurolithiatic effects were investigated using a model induced by administration of 0.75% ethylene glycol in drinking water for 28 days. The ethanolic extract exhibited reported diuretic and antiurolithiatic activity under the investigated conditions. However, increased urine output and changes in urinary biochemical parameters do not independently establish prevention or dissolution of urinary calculi. Furthermore, the specific renal molecular targets responsible for the observed effects were not identified.

Diuretic activity has also been reported for *A. hirtum*. Nalawade and Shirote [171] investigated a methanolic leaf extract administered at 150, 300, and 450 mg/kg in Wistar rats using a modified Lipschitz test. Urine output increased across the tested doses, reaching 9.70 mL at 450 mg/kg compared with 1.61 mL in controls. The highest dose produced a reported Lipschitz index of 2.35, accompanied by increased urinary sodium, potassium, and chloride excretion. These findings suggest dose-associated diuretic and saluretic activity, although the specific renal transport pathways and potential consequences of repeated electrolyte loss remain uncharacterized.

Collectively, the available investigations provide preliminary in vivo evidence of diuretic activity in selected *Abutilon* preparations. However, increased urine volume alone does not establish a specific diuretic mechanism, and changes in electrolyte excretion require interpretation alongside fluid balance and renal function. Importantly, the antiurolithiatic findings reported for *A. indicum* [170] represent a distinct pharmacological endpoint and should not be equated with clinical stone-preventive or stone-dissolving efficacy.

The available studies are further limited by incomplete chemical standardization, differences in plant organs and extraction solvents, insufficient pharmacokinetic characterization, and limited direct assessment of renal molecular targets. Although changes in sodium and potassium excretion may suggest modulation of renal electrolyte handling, involvement of specific nephron transporters remains hypothetical.

Future studies should prioritize authenticated and chemically standardized preparations, controlled dose–response experiments, comprehensive electrolyte-balance assessment, direct evaluation of renal transporter function, and comparison with established reference diuretics. Repeated-dose safety, renal histopathology, and pharmacokinetic studies are also required to assess translational relevance. Overall, the available evidence supports preliminary diuretic potential of *A. indicum* and *A. hirtum*, together with experimental antiurolithiatic activity of *A. indicum*, but remains insufficient to establish clinical efficacy or long-term renal safety.

### 7.12. Additional Cytotoxicity Evidence

Additional investigations have evaluated the cytotoxic effects of *Abutilon*-derived extracts, solvent fractions, and plant-mediated nanomaterials using cancer cell lines and preliminary toxicity-screening models. These studies provide heterogeneous evidence of biological activity and require a clear distinction between general cytotoxicity, antiproliferative effects, genotoxicity, and selective anticancer potential.

In *A. indicum*, manganese oxide (MnO) nanoparticles synthesized using a leaf extract demonstrated concentration-dependent cytotoxicity against HeLa cervical cancer cells in an MTT assay over a concentration range of 1–120 µg/mL [172]. At the highest concentration, more than 50% cell death was reported after 24 h. However, the observed effects cannot be attributed exclusively to plant-derived phytochemicals because nanoparticle composition, size, surface characteristics, and metal-associated toxicity may contribute to the cellular response.

A flavonoid-rich leaf extract of *A. indicum* was evaluated against SK-LU-1 lung, HepG2 liver, and MKN7 gastric cancer cells using the sulforhodamine B assay [173]. The reported IC_50_ values were 148.29 ± 4.55, 164.39 ± 6.94, and 164.76 ± 7.73 µg/mL, respectively. These findings indicate measurable cytotoxicity under the tested conditions, although selectivity against non-malignant cells was not established. In a separate brine shrimp lethality assay, the chloroform fraction of a methanolic extract exhibited an LC_50_ of 1.51 µg/mL [174]. This result indicates substantial lethality in a preliminary toxicity-screening model but does not demonstrate selective activity against mammalian cancer cells.

Cytotoxicity has also been reported for other *Abutilon* species. An ethyl acetate fraction obtained from a 70% ethanolic extract of the aerial parts of *A. fruticosum* inhibited H1299 lung carcinoma and HepG2 hepatocellular carcinoma cells in a 48 h sulforhodamine B assay, with IC_50_ values of 63.62 ± 0.05 and 102 ± 0.6 µg/mL, respectively [175]. The corresponding butanol fraction showed lower activity, with IC_50_ values of 294 ± 0.17 and 250 ± 0.30 µg/mL. Although fractionation enabled the identification of several phenolic constituents, their individual contributions to cytotoxicity were not directly validated in these cancer cell models.

In *A. pannosum*, a chloroform fraction demonstrated antiproliferative activity against MCF-7 breast cancer cells, with a reported IC_50_ of 50 µg/mL [176]. The effects were accompanied by G1-phase cell-cycle arrest and apoptosis-associated changes, including increased Bax and caspase-7 expression and reduced Bcl-2 expression. These findings provide additional mechanistic information beyond cell-viability measurements, although the specific active constituents and direct molecular targets remain unidentified.

In contrast, an aqueous leaf extract of *A. pannosum* produced cytotoxic and genotoxic responses in the *Allium cepa* root-tip assay [177]. The reported EC_50_ was 7.2 mg/mL, and treatment was associated with reduced mitotic activity, inhibited root growth, chromosomal abnormalities, and changes in ISSR-PCR profiles. These observations are primarily relevant to preliminary genotoxicity and safety assessment rather than selective anticancer efficacy.

Silver nanoparticles synthesized using an aqueous whole-plant extract of *A. hirtum* were evaluated against HepG2 hepatocellular carcinoma cells using a 24 h MTT assay [178]. The nanoparticles, with reported diameters of approximately 15–30 nm, were tested at concentrations of 6–200 µg/mL and exhibited greater cytotoxicity than the parent extract. The authors reported IC_50_ values of 50.78 ± 3.72 µM for the nanoparticles and 73.24 ± 6.62 µM for the extract. However, the use of molar units for nanoparticle IC_50_ values, despite mass-based exposure concentrations, was not adequately explained. This inconsistency limits quantitative interpretation and comparison with other studies. Moreover, the contribution of silver-associated toxicity and nanoparticle physicochemical properties remains unresolved.

Collectively, these studies demonstrate preliminary cytotoxic and antiproliferative activity in selected *Abutilon* preparations, but their pharmacological relevance varies substantially among experimental systems. Differences in plant material, extraction procedures, fractionation methods, nanoparticle characteristics, cell lines, exposure durations, and assay endpoints prevent reliable direct comparison of IC_50_ and LC_50_ values. Importantly, the brine shrimp lethality and *Allium cepa* assays provide preliminary toxicity or genotoxicity information and should not be interpreted as evidence of selective anticancer activity.

Studies incorporating cell-cycle analysis and apoptosis-associated markers [176] provide more mechanistically informative observations than viability assays alone. Nevertheless, changes in Bax, Bcl-2, or caspase expression do not establish direct molecular target engagement. Similarly, nanoparticle-associated cytotoxicity cannot be attributed solely to botanical constituents without appropriate controls separating the effects of the plant extract, inorganic material, and combined preparation.

A major limitation of the available literature is the insufficient evaluation of non-malignant mammalian cells and the absence of reliable selectivity indices. The genotoxic responses reported in the *Allium cepa* model [177] also warrant additional investigation in mammalian systems. Future studies should prioritize chemically characterized fractions, standardized nanoparticle characterization, appropriate material controls, concentration–response assessment, comparative cytotoxicity against non-malignant cells, direct mechanistic validation, and comprehensive genotoxicity and safety evaluation.

Overall, the available evidence supports preliminary cytotoxic and antiproliferative potential of selected *Abutilon*-derived preparations but remains insufficient to establish selective anticancer efficacy or clinical therapeutic potential.

### 7.13. Gastroprotective and Antiulcer Activities

Gastroprotective and antiulcer activities have been investigated in *A. theophrasti* and *A. indicum* using experimental models of gastric mucosal injury. The available studies report reductions in ulcer formation, changes in gastric secretory parameters, attenuation of oxidative stress, and modulation of inflammation-associated signaling. However, the biological relevance and methodological strength of these findings vary according to extract characterization, experimental design, and the extent of mechanistic validation.

Guo et al. [72] investigated a standardized aqueous whole-herb extract of *A. theophrasti* (ATAE) in a hydrochloric acid/ethanol-induced gastric ulcer model in Wistar rats and lipopolysaccharide (LPS)-stimulated RAW264.7 macrophages. UPLC-MS analysis tentatively identified 26 constituents, while rutin was quantified at 0.38% of the extract. Oral intragastric administration of ATAE at 50 and 100 mg/kg attenuated gastric mucosal injury, reduced the ulcer index and histopathological scores, and improved mucosal glycoprotein preservation, as assessed by periodic acid–Schiff staining. Treatment also reduced gastric TNF-α, IL-1β, IL-6, and malondialdehyde (MDA) levels while increasing superoxide dismutase (SOD) and glutathione peroxidase (GSH-Px) activities. Western blot analysis demonstrated reduced phosphorylation of Akt, IκBα, and NF-κB p65 in gastric tissues. In LPS-stimulated RAW264.7 macrophages, ATAE at 50 and 100 µg/mL reduced nitric oxide (NO) and reactive oxygen species (ROS) production, while additional analyses indicated reduced NF-κB p65 nuclear translocation and modulation of inflammation-associated gene expression. These complementary findings support the involvement of oxidative stress and Akt/NF-κB-associated inflammatory responses in the observed gastroprotection. However, the direct molecular targets and causal contributions of individual phytochemical constituents remain unconfirmed.

Antiulcer activity has also been reported for *A. indicum*. Aladakatti and Vijayakumar [108] evaluated an ethanolic flower extract at 250 and 500 mg/kg in an ethanol-induced gastric ulcer model in Wistar rats, reporting improvements in ulcer index, gastric acidity, and pH, particularly at the higher dose. GC-MS profiling and molecular docking against human carbonic anhydrase I and II provided additional chemical and computational information; however, these analyses did not establish direct enzyme inhibition or the molecular mechanism of gastroprotection. In a separate investigation, Dashputre and Naikwade [179] evaluated a methanolic leaf extract at 250 and 500 mg/kg in pylorus-ligation and ethanol-induced gastric ulcer models. Treatment reduced gastric secretion volume, total and free acidity, and ulcer formation, while gastric pH increased significantly at 500 mg/kg. However, inconsistencies between the reported ulcer indices and calculated protection percentages in the original study limit quantitative interpretation. Collectively, these findings provide preliminary in vivo evidence of gastroprotective activity, but the active constituents and causal molecular mechanisms remain insufficiently established.

Ponnudurai et al. [180] investigated a 90% ethanolic leaf extract of *A. indicum* in an aspirin plus pylorus ligation-induced gastric ulcer model in Wistar rats. Oral administration at 100 and 200 mg/kg reduced the ulcer score from 25.50 in ulcer controls to 12.33 and 6.50, respectively, compared with 5.33 for ranitidine at 50 mg/kg. At 200 mg/kg, gastric volume decreased from 4.28 to 2.56 mL/100 g, total acidity decreased from 78.05 to 60.16 mEq/L/100 g, and gastric pH increased from 1.62 to 3.90. Changes in mucosal carbohydrate-related parameters, including hexose, hexosamine, fucose, and sialic acid, were also reported, suggesting possible preservation of gastric mucosal protective factors. However, these functional and biochemical observations do not establish direct molecular mechanisms or confirm protection against chronic ulcer disease.

Collectively, the available studies provide complementary but methodologically heterogeneous evidence of gastroprotective activity. The investigation of *A. theophrasti* [72] offers comparatively stronger mechanistic support because macroscopic gastric injury outcomes were assessed alongside inflammatory cytokines, oxidative stress biomarkers, and signaling-related protein measurements. In contrast, the *A. indicum* studies [108,179,180] primarily demonstrate reductions in ulcer severity and changes in gastric secretory or mucosal parameters. Although GC-MS profiling and molecular docking were additionally performed in one investigation [108], these approaches do not establish direct molecular target engagement or a causal mechanism of gastroprotection. Furthermore, inconsistencies between reported ulcer indices and protection percentages in another study [179] limit the reliability of quantitative efficacy estimates. Differences in plant organs, extraction solvents, dosing regimens, and ulcer-induction procedures further prevent reliable direct comparisons of gastroprotective potency across studies.

An important translational limitation is that acute chemically induced gastric injury and pylorus-ligation models do not fully reproduce the multifactorial pathophysiology of human peptic ulcer disease, including *Helicobacter pylori* infection, chronic mucosal inflammation, and recurrent ulceration. Furthermore, most preparations lack comprehensive phytochemical standardization, pharmacokinetic characterization, and repeated-dose safety evaluation. The association of Akt/NF-κB signaling changes with gastroprotection [72] also requires direct mechanistic validation to establish causality.

Future investigations should prioritize authenticated and chemically standardized extracts, controlled dose–response studies, isolation and testing of active constituents, direct molecular target validation, and complementary models of chronic gastric injury. Pharmacokinetic studies and comprehensive safety assessments are also necessary before clinical translation. Overall, the available evidence supports preliminary gastroprotective and antiulcer potential of selected *Abutilon* preparations, with comparatively stronger mechanistic evidence for *A. theophrasti*, but remains insufficient to establish clinical efficacy or long-term safety.

### 7.14. Anti-Arthritic Activity

The potential anti-arthritic activity of *A. indicum* has been investigated using an experimental model of adjuvant-induced arthritis and an in vitro cyclooxygenase-2 (COX-2) inhibition assay. These studies provide complementary but methodologically distinct evidence of anti-inflammatory and anti-arthritic-related activity, although their pharmacological relevance depends on the experimental model and the degree of extract characterization.

Bhajipale [181] evaluated a methanolic extract prepared from the aerial parts of *A. indicum* in Wistar rats with complete Freund’s adjuvant-induced arthritis. The extract was administered orally at 100, 200, and 400 mg/kg, with methotrexate (0.75 mg/kg) as a reference treatment. At 400 mg/kg, the extract significantly reduced paw volume on days 7 and 14 (*p* < 0.01), whereas the lower doses did not produce statistically significant reductions. Mean paw volume at 400 mg/kg was 1.842 mL on day 7 and 1.725 mL on day 14, compared with 2.102 and 2.005 mL, respectively, in untreated arthritic controls. The methotrexate group showed corresponding values of 1.716 and 1.601 mL. Qualitative observations also suggested reductions in secondary lesions and improvements in radiographic joint appearance at the highest extract dose. However, these observations were not supported by standardized quantitative assessments of bone erosion or cartilage damage, limiting conclusions regarding structural joint protection.

Cinthura et al. [182] investigated the COX-2 inhibitory activity of a commercially obtained *A. indicum* extract using an in vitro enzyme inhibition assay. The extract exhibited concentration-dependent inhibition over 15.625–1000 µg/mL, with a reported IC_50_ of 116.6 µg/mL and maximum inhibition of 95.33% at 1000 µg/mL. These findings provide preliminary biochemical evidence of COX-2 inhibition. However, the plant material and extraction solvent were not specified, and neither COX-1 selectivity nor cellular target engagement was established. Furthermore, the preparation differed from the methanolic extract used in the animal study [181], preventing direct attribution of the observed anti-arthritic effects to COX-2 inhibition.

Collectively, the available studies support preliminary anti-inflammatory and anti-arthritic-related activity of *A. indicum*, but the strength of evidence differs substantially between the experimental models. The adjuvant-induced arthritis model [181] provides greater physiological relevance than a cell-free enzyme assay because it incorporates systemic inflammatory responses and joint-associated pathological changes. Nevertheless, reduced paw swelling does not independently establish disease-modifying activity, and qualitative radiographic findings require confirmation through standardized histopathological and structural assessments.

The COX-2 inhibition reported in vitro [182] offers a plausible biochemical basis for anti-inflammatory activity but does not establish suppression of the autoimmune mechanisms underlying rheumatoid arthritis. Moreover, the absence of chemical standardization, direct target validation in relevant cellular systems, and reliable COX-1/COX-2 selectivity data limits mechanistic interpretation. No causal relationship between COX-2 inhibition and reduced arthritic paw swelling has been demonstrated.

Future investigations should prioritize authenticated and chemically standardized preparations, isolation and characterization of active constituents, controlled dose–response experiments, COX-1/COX-2 selectivity assessment, cellular target validation, quantitative evaluation of joint pathology, inflammatory cytokine profiling, and comprehensive pharmacokinetic and repeated-dose safety studies. Overall, the available evidence supports preliminary anti-arthritic potential of *A. indicum*, particularly at 400 mg/kg in an experimental arthritis model, but remains insufficient to establish disease-modifying efficacy, clinical benefit, or equivalence to established antirheumatic therapies.

### 7.15. Enzyme-Inhibiting Activity

Enzyme-inhibitory activity of *A. indicum* has been investigated using in vitro biochemical assays targeting pepsin and α-glucosidase. Although both investigations reported measurable enzyme inhibition, their biological relevance and pharmacological interpretation differ substantially because of differences in enzyme targets, extract characterization, and experimental conditions.

Budhanale et al. [183] reported 40.81% inhibition in a pepsin-based biochemical assay at an extract concentration of 1 mg/mL. However, this assay does not directly assess HIV protease inhibition or antiviral activity, and the absence of target-specific validation substantially limits the interpretation of these findings.

Yaisaeng et al. [76] investigated the α-glucosidase inhibitory activity of a hexane root extract of *A. indicum* and its silica gel chromatographic fractions. The crude extract (RAIH) exhibited 88.06% inhibition at the reported concentration of 500 mg/mL, whereas fractions RAIH2, RAIH3, and RAIH4 produced inhibition values of 93.55%, 95.19%, and 93.81%, respectively. Fraction RAIH1 showed 75.81% inhibition, while RAIH5–RAIH7 were inactive under the investigated conditions. These results indicate that inhibitory activity was concentrated in selected chromatographic fractions, although the absence of concentration–response data prevents reliable comparison of inhibitory potency.

Chemical investigation of the fractions led to the isolation of four sterol-type constituents: β-sitosterol, stigmasterol, daucosterol, and stigmasterol-3-O-β-D-glucoside [76]. However, these isolated compounds were not individually evaluated for α-glucosidase inhibition in the same experimental system. Their possible contribution to the observed fraction activity was inferred from previously published findings and molecular docking predictions. Consequently, the association between these constituents and α-glucosidase inhibition remains provisional rather than directly demonstrated.

In *A. figarianum*, Saleem et al. [78] additionally reported tyrosinase-inhibitory activity for methanolic and dichloromethane whole-plant extracts, with values of 129.01 ± 0.97 and 112.89 ± 2.16 mg KAE/g extract, respectively. Although the methanolic extract exhibited a higher inhibitory response, these kojic acid-equivalent measurements do not establish the inhibitory potency of individual constituents or demonstrate effects on melanogenesis in cellular systems. Therefore, the findings remain preliminary biochemical evidence rather than validation of a dermatological therapeutic application.

Collectively, these studies provide preliminary biochemical evidence of pepsin and α-glucosidase inhibition by *A. indicum* preparations and tyrosinase inhibition by *A. figarianum* extracts [78], but do not establish therapeutic efficacy or direct molecular target engagement in biological systems. In the pepsin-based assay, the *A. indicum* extract exhibited 40.81% inhibition at 1 mg/mL, compared with 93.87% inhibition by pepstatin at 100 µg/mL [183]. However, this comparison involves different concentrations and preparations and does not establish relative inhibitory potency or HIV-specific antiviral activity. In contrast, the α-glucosidase investigation [76] incorporated bioassay-guided chromatographic fractionation and structural identification of sterol-type constituents, providing more chemically informative evidence. Nevertheless, the reported testing concentration of 500 mg/mL is unusually high, and the distinction between stock-solution and final assay concentrations is insufficiently clear, raising concerns regarding assay interference and pharmacologically achievable exposure. Furthermore, the isolated constituents were not individually tested for α-glucosidase inhibition in that study; their reported IC_50_ values were derived from the previous literature rather than directly measured. Accordingly, the observed fraction-level inhibition cannot be attributed to specific isolated compounds without further experimental validation.

Future investigations should prioritize chemically standardized preparations, concentration–response characterization, direct testing of purified constituents, appropriate reference inhibitors, and evaluation of nonspecific enzyme inhibition or assay interference. Pharmacokinetic and safety studies are also required before therapeutic relevance can be assessed. Overall, the available findings support preliminary enzyme-inhibitory activity of selected *A. indicum* preparations, particularly α-glucosidase inhibition by non-polar root fractions, together with tyrosinase-inhibitory activity of *A. figarianum* extracts [78]. However, insufficient concentration–response characterization, limited constituent-level validation, and the absence of relevant cellular or in vivo confirmation prevent conclusions regarding pharmacologically relevant potency or clinical efficacy.

### 7.16. Aphrodisiac Activity

Aphrodisiac and reproductive effects of *A. indicum* and *A. pannosum* have been investigated in preclinical animal models assessing sexual behavior, erectile responses, sperm parameters, and reproductive hormone levels. Although several favorable changes have been reported, the biological relevance of these findings varies according to the experimental endpoints and the degree of botanical and mechanistic characterization.

Ganu et al. [184] investigated an aqueous root extract of *A. indicum* in experimental models of sexual behavior and reproductive function. Oral administration at 100, 200, and 400 mg/kg was evaluated in Swiss mice and Wistar rats. At 200 and 400 mg/kg, the extract increased mounting frequency, penile erection episodes, and mating-related responses. The penile erection index increased from 25 in controls to 229 and 332 at 200 and 400 mg/kg, respectively, compared with 350 for sildenafil at 5 mg/kg. Following 28 days of treatment, sperm count increased from 121.5 ± 2.39 in controls to 144.88 ± 3.77 in the 400 mg/kg group. In isolated rat corpus cavernosum preparations, the extract at 10 µg/mL produced relaxation in the presence of the nitric oxide donor sodium nitroprusside, with residual contraction of 51 ± 3.1% after 15 min. Although these observations suggest possible involvement of nitric oxide-associated smooth-muscle responses, direct phosphodiesterase inhibition, intracellular cGMP accumulation, and the causal contribution of these processes were not experimentally demonstrated.

In *A. pannosum*, Khalil et al. [185] evaluated a methanolic stem-bark extract administered to male rats at 400 mg/kg daily for 5, 10, and 15 days. Treatment reduced mount latency, intromission latency, and the post-ejaculatory interval, while increasing erection index and selected copulatory performance measures. After 15 days, mount, intromission, and ejaculation frequencies were significantly increased compared with controls (*p* < 0.05). The proportions of viable and motile spermatozoa also increased after 10 and 15 days, whereas the increase in total sperm count was not statistically significant. Serum testosterone levels and the weights of selected reproductive organs also increased following treatment. These findings suggest concurrent behavioral and reproductive responses, although the active constituents and endocrine mechanisms responsible for the observed effects remain unidentified.

Collectively, these investigations provide preliminary in vivo and ex vivo evidence of aphrodisiac-related activity in selected *Abutilon* preparations. However, sexual behavior, erectile responses, sperm quality, and reproductive hormone levels represent distinct endpoints and should not be interpreted as equivalent evidence of improved fertility. The *A. indicum* study [184] provides complementary behavioral and functional observations, but the smooth-muscle relaxation was assessed in the presence of an exogenous nitric oxide donor, preventing confirmation of a specific NO–cGMP-dependent mechanism. Similarly, the hormonal and sperm-quality changes reported for *A. pannosum* [185] do not establish androgen receptor activation or improved reproductive outcomes.

The available evidence is further limited by incomplete chemical standardization, insufficient identification of active constituents, differences in dosing regimens, and the absence of comprehensive reproductive safety assessments. Furthermore, direct comparisons between the two studies are constrained by differences in plant organs, extraction solvents, animal models, and experimental endpoints.

Future investigations should prioritize chemically standardized preparations, controlled dose–response experiments, direct assessment of phosphodiesterase activity and intracellular cGMP, reproductive hormone profiling, validated fertility outcomes, pharmacokinetic characterization, and repeated-dose reproductive toxicity studies. Overall, the available findings support preliminary aphrodisiac and reproductive effects of *A. indicum* and *A. pannosum* in experimental animals but remain insufficient to establish clinical efficacy, improved fertility, or reproductive safety in humans.

### 7.17. Other Reported Pharmacological Activities

Several additional pharmacological activities have been investigated in *Abutilon* species, including anthelmintic, immunomodulatory, antidiarrheal, hormone-modulating, antimycobacterial, wound-healing, antivenom-related, antiallergic, antiparasitic, antimalarial, nephroprotective, pulmonoprotective, and anticataract effects. Most of these activities are supported by individual preclinical investigations rather than independently replicated studies, and their interpretation requires consideration of experimental relevance, quantitative outcomes, and methodological limitations.

Hassan et al. [186] evaluated methanolic stem extracts of *A. theophrasti* against *Haemonchus contortus*, reporting 74.39% egg-hatch inhibition and 79.79% larval-motility inhibition at 500 mg/mL, compared with 98.6% and 92.88%, respectively, for levamisole at 2 mg/mL. The substantially higher extract concentration and absence of in vivo validation limit conclusions regarding anthelmintic potency. Immunomodulatory effects were subsequently investigated using aqueous and ethanolic leaf extracts of *A. indicum* [187]. The ethanolic preparation at 200 mg/kg increased the reported phagocytic index to 0.076 compared with 0.029 in controls, whereas the aqueous preparation at 400 mg/kg yielded a lower value of 0.017. These contrasting responses indicate preparation-dependent immunomodulation rather than uniformly stimulatory activity.

Antidiarrheal effects were reported for *A. indicum* leaf extracts in castor oil-induced diarrhea and complementary intestinal models [188]. At 500 mg/kg, methanolic and aqueous extracts reduced fecal output to 10.25 ± 0.22 and 15.38 ± 0.53 g/kg, respectively, compared with 23.52 ± 0.34 g/kg in controls. However, the underlying intestinal molecular mechanisms were not established. In a separate investigation, a 50% methanolic fruit extract of *A. indicum* exhibited predominantly antiestrogenic responses under estradiol-stimulated conditions [189]. At 500 mg/kg, estradiol-stimulated uterine weight decreased from 77.8 to 33.2 mg/100 g body weight. These findings suggest hormone-modulating activity but do not demonstrate direct estrogen receptor binding or subtype-specific signaling.

Additional screening investigations have examined antimycobacterial, wound-healing, and antivenom-related activities. An *A. indicum* leaf preparation was reported to inhibit *Mycobacterium tuberculosis* growth at concentrations of 12.5–100 µg/mL [183], although incomplete methodological reporting limits confidence in the reproducibility of this finding. A 5% extract-containing ointment of *A. indicum* was associated with progressive wound closure in experimental rat models, with complete closure reported by day 16 [190]. However, specific wound-repair mechanisms and the comparative contributions of different extracts were not established. A methanolic leaf extract also inhibited several *Echis carinatus* venom-associated enzymes at 250 µg/mL [191]. Nevertheless, biochemical enzyme inhibition cannot be equated with systemic venom neutralization, and protective efficacy in appropriate animal models remains unverified.

Antiallergic activity has been investigated using a 70% hydroethanolic aerial-part extract of *A. indicum* and selected constituents [192]. In ionomycin-stimulated RBL-2H3 cells, the preparations reduced β-hexosaminidase and histamine release, while the extract produced 70.88% protection against compound 48/80-induced rat mesenteric mast-cell degranulation at 400 µg/mL. However, responses of individual constituents were not uniformly concentration-dependent, and molecular docking predictions involving cysteinyl leukotriene receptor-1 did not establish direct receptor engagement. Antiparasitic screening of *A. pannosum* demonstrated 79% inhibition of *Giardia lamblia* trophozoites at 500 µg/mL, with an antigiardial IC_50_ of 89.7 µg/mL and a Vero-cell cytotoxicity IC_50_ of 279.94 µg/mL [114]. These results indicate the need for further selectivity assessment. In *A. hirtum*, a chloroform leaf fraction produced 78% initial inhibition of *Leishmania donovani* intracellular amastigotes at 20 µg/mL, but the follow-up IC_50_ exceeded 20 µg/mL [193], limiting conclusions regarding inhibitory potency.

Comparatively broader preclinical evidence has been reported for antimalarial and organ-protective activities. Ethyl acetate fractions of *A. grandiflorum* root-derived preparations inhibited *Plasmodium falciparum*, with reported IC_50_ values of 9–14 µg/mL [194]. In *P. vinckei*-infected mice, selected fractions reduced parasitemia and prolonged survival; however, experimental groups contained only two or three animals, substantially limiting statistical reliability. In a separate investigation, a partially chemically characterized methanolic stem-bark extract of *A. pannosum* administered at 200 and 400 mg/kg attenuated CCl_4_-induced kidney and lung injury in rats [113]. Improvements were reported in antioxidant-associated parameters, inflammatory responses, histopathological findings, and DNA-damage measurements. Although these complementary endpoints provide broader biological support than isolated screening assays, the findings remain restricted to a chemically induced injury model and do not identify the individual constituents responsible for the observed protection.

Anticataract activity was investigated using a hydroethanolic leaf extract of *A. indicum* in an ex vivo glucose-induced cataract model involving isolated goat lenses [195]. At 500 µg/mL, treatment increased lens protein content from 2.2 to 8.8 mg/mL and superoxide dismutase activity from 3.11 to 7.81 units/mg tissue, while reducing the reported malondialdehyde value from 0.0026 to 0.0021. Reduced lens opacity and aldose reductase-related responses were also reported. However, these findings do not establish a direct molecular mechanism or demonstrate efficacy in in vivo cataract models.

Collectively, these less-explored activities illustrate the breadth of preliminary pharmacological investigations involving *Abutilon*, but the strength of evidence varies substantially. Antimalarial [194] and nephroprotective/pulmonoprotective [113] investigations incorporated complementary biological outcomes, whereas anthelmintic [186], antimycobacterial [183], and antivenom-related [191] findings relied mainly on preliminary screening methods. Preparation-dependent immunomodulatory responses [187], limited antiparasitic selectivity [114,193], and the absence of direct mechanistic validation further constrain interpretation. Across these investigations, differences in botanical materials, extract composition, experimental models, concentrations, and reference controls preclude reliable cross-study potency comparisons.

Future studies should prioritize chemically standardized preparations, reproducible concentration–response experiments, appropriate reference treatments, direct molecular target validation, pharmacokinetic characterization, and preparation-specific safety assessment. Promising findings should be replicated in complementary biological models before progression toward clinical investigation. Overall, these additional pharmacological activities remain predominantly preliminary and do not establish clinical therapeutic efficacy.

**Table 3:** Critical comparative summary of selected pharmacological evidence for *Abutilon* species.

Species [Ref.]	Preparation and Experimental Model	Dose/Concentration	Principal Findings	Evidence Strength and Limitations
Antioxidant Activity				
*A. indicum* [109]	Ethanolic extract; CCl_4_-induced oxidative stress in rats	100, 300, 500 mg/kg, p.o.	Improved hepatic GSH, SOD, catalase, and peroxidase parameters, with the greatest reported effects at 500 mg/kg	In vivo; animal oxidative stress model; no human efficacy data.
*A. figarianum* [78]	Methanolic and dichloromethane whole-plant extracts; DPPH, ABTS, FRAP, CUPRAC, phosphomolybdenum, and ferrous-ion chelation assays	Assay concentrations not specified in the main article; protocols in Supplementary Materials	Methanolic extract: DPPH 66.64, ABTS 120.92, FRAP 123.16, CUPRAC 204.26 mg TE/g; DCM extract: metal chelation 51.57 mg EDTAE/g.	Cell-free chemical assays; no cellular or in vivo validation.
Anti-inflammatory Activity				
*A. indicum* [124]	Ethanolic leaf extract; Isoniazid-induced inflammatory injury in rats	250, 500 mg/kg	Reduced TNF-α and NF-κB gene expression; increased IκB expression	In vivo; gene-expression changes; protein-level pathway causality not established.
*A. theophrasti* [125]	Total flavonoid leaf fraction; LPS-induced acute lung injury in mice	0.25, 0.5, 1.0 g/kg	Reduced inflammatory mediators, iNOS/COX-2 expression and NF-κB/MAPK-associated signaling responses	In vivo; pathway-associated protein responses; direct target engagement unverified.
Antimicrobial Activity				
*A. theophrasti* [135]	Isolated hibicuslide C; *P. aeruginosa* ATCC 27853 and six multidrug-resistant clinical isolates	MIC = 5–10 µg/mL	Growth inhibition, DNA fragmentation, reduced biofilm biomass, and antibiotic-combination effects	In vitro; bacterial susceptibility and biofilm endpoints; in vivo efficacy untested.
*A. hirtum* [142]	Crude extract and AF9 fraction; Multidrug-resistant *Salmonella* isolates	MIC = 150–300 µg/mL; MBC = 300–600 µg/mL	Antibacterial activity against resistant isolates	In vitro; multidrug-resistant isolates; clinical relevance unverified.
*A. bidentatum* [34]	Isolated abubidentin A; *E. coli*, *P. aeruginosa*, *S. aureus*	Reported MIC ≤ 150, ≤125 and ≤125 µg/mL	Growth inhibition against all three tested bacterial species	In vitro; isolated-compound susceptibility testing; in vivo efficacy untested.
Hepatoprotective Activity				
*A. indicum* + *P. longum* [85]	Combined-extract phytosomal formulation; CCl_4_-induced hepatotoxicity in rats	Phytosome 100 mg/kg; combined extract 100, 200, 400 mg/kg	Improved biochemical and histopathological outcomes; individual species contributions unresolved	In vivo; combined-species phytosomal formulation; individual contributions unresolved.
*A. indicum* [149]	Hydroalcoholic stem-extract tablet; Paracetamol-induced hepatotoxicity in rats	200 mg/kg (abstract); 250 mg/kg (discussion/conclusion)	Improvements in biochemical markers of liver injury	In vivo; tablet formulation; administered dose inconsistently reported.
Antidiabetic Activity				
*A. indicum* [7]	Aqueous whole-plant extract and butanol fraction; Diabetic rats; 3T3-L1 adipocytes; transcriptional reporter assays	Butanol fraction: 100 µg/mL in cellular assays	Reduced postprandial glucose; PPARγ-associated responses and GLUT1 promoter activation	In vivo and in vitro; reporter activation does not establish functional GLUT1 upregulation.
*A. indicum* [153]	Methanolic leaf extract; α-Amylase and α-glucosidase inhibition assays	10–160 µg/mL	Reported IC_50_ = 129.89 and 137.61 µg/mL, respectively; maximum observed inhibition below 50%, limiting reliability	In vitro biochemical assays; reported IC50 values inconsistent with inhibition below 50%.
*A. indicum* [155]	Aqueous extract; Diabetic rats; intestinal glucose absorption and insulin-secretion models	0.5–1 g/kg in rats	Reduced postprandial glucose; altered intestinal glucose absorption and insulin secretion	In vivo, ex vivo and in vitro; relative causal contributions not established.
Antitumor Activity				
*A. indicum* [160]	Polyphenol-stabilized gold nanoparticles; HT-29 colorectal cancer cells	IC_50_ = 210 µg/mL (24 h); 180 µg/mL (48 h)	Cytotoxicity associated with ROS generation, mitochondrial dysfunction, cell-cycle arrest, and apoptosis-related markers	In vitro; nanomaterial-associated cytotoxicity; tumor selectivity not established.
*A. bidentatum* [91]	Ethanolic aerial-part extract; Sarcoma-180 tumor model	100 mg/kg/day	Tumor growth ratio 83.2%; classified as negative for antitumor activity in the original study	In vivo; negative antitumor outcome reported in the original study.
Neurotropic and Neuroprotective Activities				
*A. indicum* [162]	Methanolic whole-plant extract; AlCl_3_-induced cognitive impairment in Sprague–Dawley rats; elevated plus maze, radial arm maze, passive avoidance and brain AChE measurements	400, 600 mg/kg, p.o.; AlCl_3_ 4.2 mg/kg, i.p., 28 days	Improved selected memory-related behavioral outcomes; brain AChE activity decreased from 1.898 in model controls to 0.783 and 0.635 in treated groups; behavioral dose–response was not uniform	In vivo; behavioral dose–response not uniform.
Anticonvulsant Activity				
*A. indicum* [164]	Chloroform, 90% ethanolic and aqueous stem extracts; MES- and PTZ-induced seizures in Wistar rats (*n* = 6/group)	200 mg/kg, p.o.	Chloroform extract abolished tonic hind-limb extension in MES and prevented observed PTZ-induced convulsions in all six treated rats; ethanolic and aqueous extracts showed weaker effects	In vivo; small experimental groups (*n* = 6); clinical efficacy unestablished.
Anti-Asthmatic Activity				
*A. indicum* [166]	Powdered dried aerial parts; open-label, uncontrolled clinical study; 30 completers with mild-to-moderate bronchial asthma	1 g orally, three times daily for 4 weeks	FVC increased from 1.61 to 1.86 L and FEV_1_ from 1.30 to 1.48 L; symptoms and MVV improved; FEV_1_/FVC and FEF_25–75%_ changes were not significant	Uncontrolled clinical study (30 completers); treatment effect cannot be causally attributed.
*A. indicum* [167]	Methanolic aerial-part extract; Guinea-pig bronchospasm, isolated smooth-muscle contraction, rat mast-cell degranulation and paw-edema models	250–500 mg/kg, p.o.; 250–1000 µg/mL (isolated tissue); 10–33.33 mg/mL (mast-cell assays)	Mast-cell stabilization and reduced paw edema; no significant direct bronchodilation or inhibition of isolated smooth-muscle contraction	In vivo, ex vivo and in vitro; direct bronchodilation not demonstrated.
Additional Cytotoxicity Evidence				
*A. pannosum* [177]	Aqueous leaf extract; Allium cepa root-tip cytogenetic assay	EC_50_ = 7.2 mg/mL	Reduced mitotic index, inhibited root growth and chromosomal abnormalities	Plant genotoxicity bioassay; mammalian safety not established.
Gastroprotective and Antiulcer Activities				
*A. theophrasti* [72]	Standardized aqueous whole-herb extract (ATAE); rutin 0.38%; HCl/EtOH-induced gastric ulcer in Wistar rats; LPS-stimulated RAW264.7 cells	50, 100 mg/kg, i.g.; 50, 100 µg/mL in vitro	Reduced ulcer index, inflammatory cytokines and MDA; increased SOD and GSH-Px; reduced Akt/IκBα/NF-κB p65 phosphorylation and cellular ROS/NO	In vivo and in vitro; pathway association observed, but direct target engagement unverified.
Anti-Arthritic Activity				
*A. indicum* [181]	Methanolic aerial-part extract; Complete Freund’s adjuvant-induced arthritis in Wistar rats (*n* = 6/group); paw volume, secondary lesions and radiographic assessment	100, 200, 400 mg/kg, p.o.; methotrexate 0.75 mg/kg	At 400 mg/kg, paw volume was 1.842 mL (day 7) and 1.725 mL (day 14) versus 2.102 and 2.005 mL in controls (*p* < 0.01); lower doses were not significant	In vivo; only the highest tested dose significantly reduced paw volume.
Enzyme-Inhibiting Activity				
*A. indicum* [76]	Hexane root extract and chromatographic fractions RAIH1–RAIH7; α-Glucosidase enzyme inhibition assay	500 mg/mL, as reported	Crude extract: 88.06%; RAIH2: 93.55%; RAIH3: 95.19%; RAIH4: 93.81%; RAIH5–RAIH7: inactive; isolated sterols not individually tested	In vitro biochemical assay; single-concentration screen; isolated sterols not individually tested.
Other Reported Pharmacological Activities				
*A. indicum* [192]	70% hydroethanolic aerial-part extract (HAEAI); SYD, MC, M3IC; Ionomycin-stimulated RBL-2H3 cells; compound 48/80-induced rat mesenteric mast-cell degranulation; molecular docking	In vitro: HAEAI 200, SYD 50, MC 4.45, M3IC 0.017 µg/mL; ex vivo: HAEAI 100–400, SYD 25–100, MC 2–10, M3IC 0.01–0.04 µg/mL	Reduced β-hexosaminidase and histamine release; HAEAI ex vivo protection reached 70.88% at 400 µg/mL; M3IC docking score −6.060	In vitro, ex vivo and in silico; docking predictions not experimentally validated.
*A. indicum* [195]	Hydroethanolic leaf extract; Glucose-induced cataract in isolated goat lenses; aldose reductase assay	500 µg/mL	Reduced lens opacity and MDA; increased SOD and protein content; aldose reductase inhibition	Ex vivo and in vitro; isolated-lens model; no clinical cataract efficacy evidence.

Note: This table prioritizes representative quantitative, mechanistic, clinical, formulation-related and negative findings. Evidence descriptors are qualitative and are not formal risk-of-bias scores. Additional study entries appear in Supplementary Table S1.

### 7.18. Safety and Toxicological Evidence

Safety and toxicological evidence for *Abutilon* species remains considerably less extensive than the available pharmacological literature. Most investigations have evaluated individual botanical preparations using acute toxicity tests, short-term repeated-dose studies, or preliminary cellular and genotoxicity assays. Consequently, the findings cannot be generalized across different species, plant organs, extraction systems, or formulations.

A comparatively detailed toxicological investigation was conducted by Islam et al. [98] using a methanolic seed extract of *A. indicum*. In acute oral toxicity testing, no mortality was observed in Wistar rats at doses of 250–2000 mg/kg during a 14-day observation period, indicating an oral LD_50_ above the highest tested dose of 2000 mg/kg. In a 28-day repeated-dose study, administration at 250 and 500 mg/kg was not associated with significant adverse changes in the evaluated biochemical, hematological, histopathological, or genotoxicity-related endpoints. In contrast, treatment at 1000 mg/kg significantly increased liver weight and serum ALT, AST, and ALP activities and produced mild hepatic histopathological alterations. Comet assay results additionally demonstrated increased DNA damage in both male and female rats at this dose. These findings indicate dose-associated hepatotoxic and genotoxic responses under the investigated conditions.

Complementary in vitro investigations [98] showed limited cytotoxicity toward HEK-293 cells at concentrations up to 250 µg/mL, whereas cellular viability decreased at higher concentrations. The Ames assay also indicated increased revertant colony formation at 1.5–2.5 mg/mL. However, the reported bacterial strain designation was inconsistent with standard Ames-test nomenclature, limiting confidence in the interpretation of the mutagenicity findings. Although 500 mg/kg represented the highest tested repeated dose without significant adverse findings, the small experimental groups and 28-day observation period preclude establishing a general safe dose range or long-term safety profile.

Additional acute toxicity information was reported by Sharma and Sahu [85] for a combined ethanolic extract of *A. indicum* leaves and *Piper longum* fruits (3:1). No mortality was reported during the observation period, and the oral LD_50_ was stated to exceed 5000 mg/kg in rats. Importantly, this result applies to the combined botanical extract rather than *A. indicum* alone or the phytosomal formulation developed in the same investigation. This study did not establish an independent LD_50_ for the phytosomes or provide comprehensive repeated-dose toxicity data. Therefore, the reported acute toxicity findings cannot demonstrate the long-term safety of the formulation.

Toxicological findings also vary according to botanical preparation and experimental model. An aqueous leaf extract of *A. pannosum* produced cytotoxic and genotoxic effects in the *Allium cepa* root-tip assay, including reduced mitotic activity and chromosomal abnormalities [177]. An ethanolic extract exhibited a reported cytotoxicity IC_50_ of 279.94 µg/mL in Vero cells [114]. However, these results were obtained using different experimental systems and should not be interpreted as equivalent measures of safety. In particular, the Vero-cell IC_50_ indicates measurable cytotoxicity and does not establish the absence of toxicity in mammalian tissues.

An additional limitation concerns plant-mediated nanoparticles and isolated phytochemical constituents. Their toxicological properties may depend on particle composition, size, surface chemistry, exposure duration, and interactions with biological systems. Accordingly, safety observations obtained for crude botanical extracts cannot be directly extrapolated to nanoparticle-based preparations.

Overall, the available evidence indicates preparation- and dose-dependent toxicological responses, with the most detailed repeated-dose findings currently available for the methanolic seed extract of *A. indicum* [98]. However, the limited number of studies, small experimental groups, incomplete chemical standardization, and absence of comprehensive chronic, reproductive, developmental, and herb–drug interaction assessments prevent definition of standardized safe dose ranges. Although 500 mg/kg was the highest tested repeated dose without statistically significant adverse findings in the investigated *A. indicum* seed-extract study [98], the available data are insufficient to establish a broadly applicable no-observed-adverse-effect level (NOAEL), exposure-based safety margins, or acceptable human dosing recommendations. Future investigations should integrate chemically characterized preparations, validated genotoxicity assays, pharmacokinetic assessment, repeated-dose toxicity studies, and exposure-based safety margins before clinical translation can be considered.

## 8. Molecular Mechanisms of the Pharmacological Effects of *Abutilon*

Although a broad range of pharmacological activities has been reported for *Abutilon* species, mechanistic evidence remains substantially more limited than phenotypic evidence and is concentrated mainly on *A. indicum* and *A. theophrasti*. Available investigations have primarily examined inflammatory signaling, glucose-regulatory processes, and oxidative stress-related responses. However, the interpretation of these findings requires a distinction between experimentally observed pathway modulation, biochemical enzyme inhibition, and computationally predicted molecular interactions.

### 8.1. Inflammatory Signaling

Inflammatory signaling represents one of the most extensively investigated mechanistic areas. In *A. theophrasti*, a total leaf flavonoid fraction attenuated lipopolysaccharide (LPS)-induced acute lung injury in mice, accompanied by reduced iNOS and COX-2 expression and suppression of NF-κB/MAPK-associated signaling responses [125]. These observations provide experimental evidence of changes in inflammation-associated molecular pathways, although direct interactions between individual flavonoids and specific signaling proteins were not established.

In *A. indicum*, an ethanolic leaf extract reduced TNF-α and NF-κB gene expression and increased IκB expression in an isoniazid-induced experimental model, while TGF-β responses were less consistent [124]. These findings suggest modulation of inflammatory responses under chemically induced injury conditions. However, changes in gene expression do not independently establish inhibition of NF-κB protein activation or demonstrate that the observed hepatoprotective effects were causally mediated by this pathway.

Collectively, these studies provide mechanistic context beyond conventional inflammation-screening assays, but the active constituents and direct molecular targets remain insufficiently characterized.

### 8.2. Glucose-Regulatory Mechanisms

Glucose-regulatory mechanisms have been investigated primarily in *A. indicum*. An aqueous whole-plant extract and its butanol fraction were associated with reduced postprandial hyperglycemia in diabetic rats, PPARγ-associated transcriptional responses, enhanced adipocyte differentiation, and increased GLUT1 promoter activity in cellular models [7]. However, transcriptional reporter activation does not establish increased GLUT1 protein abundance or functional transporter-mediated glucose uptake. Furthermore, direct activation of Akt/GSK-3β signaling was not demonstrated under the reported experimental conditions.

Additional investigations have reported inhibition of carbohydrate-digesting enzymes, including α-amylase and α-glucosidase [153], together with antiglycation activity [154]. However, inconsistencies between the reported IC_50_ values and observed inhibition ranges in the enzyme-inhibition study [153] limit quantitative interpretation. Similarly, the antiglycation findings [154] provide preliminary biochemical evidence but do not establish improved systemic glucose regulation.

An aqueous extract of *A. indicum* was also reported to reduce intestinal glucose absorption and stimulate insulin secretion in complementary experimental models [155]. Nevertheless, these findings do not establish the relative contributions of intestinal, pancreatic, and peripheral metabolic processes to the observed glucose-lowering effects.

Overall, the available evidence suggests that selected *A. indicum* preparations may influence several processes associated with glucose homeostasis, but a unified and causally validated molecular mechanism has not been established.

### 8.3. Gastroprotective and Oxidative Stress-Related Signaling

Gastroprotective effects have been associated with changes in inflammatory signaling and oxidative stress-related parameters. Guo et al. [72] investigated a standardized aqueous extract of *A. theophrasti* in an HCl/ethanol-induced gastric ulcer model and LPS-stimulated RAW264.7 macrophages. Treatment reduced TNF-α, IL-1β, IL-6, and malondialdehyde levels while increasing antioxidant enzyme activities, including SOD and GSH-Px. Western blot analysis further demonstrated reduced phosphorylation of Akt, IκBα, and NF-κB p65 in gastric tissues. In RAW264.7 cells, the extract reduced reactive oxygen species and nitric oxide production and attenuated NF-κB-associated inflammatory responses.

These findings provide comparatively stronger pathway-level evidence because biochemical, histopathological, and signaling-related outcomes were evaluated in complementary experimental systems. Nevertheless, changes in phosphorylation and oxidative stress biomarkers do not independently establish direct target engagement or identify the individual phytochemical constituents responsible for gastroprotection.

### 8.4. Computationally Predicted Molecular Interactions

Computational studies have explored possible interactions between *Abutilon*-derived constituents and disease-associated molecular targets. In a gastric cancer-related molecular docking investigation, myricetin from *A. theophrasti* exhibited a predicted binding energy of 8.7 kcal/mol against a protein associated with TGF-β signaling [161]. However, this computational result does not demonstrate direct target binding, pathway inhibition, or anticancer efficacy.

Similarly, molecular docking predictions involving isolated *A. indicum* constituents and inflammation-associated targets [74] provide hypotheses for subsequent experimental investigation rather than confirmed molecular mechanisms.

Computational investigations have also examined potential interactions between tentatively annotated constituents of *A. figarianum* and selected enzyme targets. Saleem et al. [78] reported predicted binding energies of 11.26 kcal/mol for 6-hydroxyluteolin 5-rhamnoside with acetylcholinesterase, 9.38 kcal/mol for 3-O-acetylhamayne with acetylcholinesterase, and 7.63 kcal/mol for isobergapten with tyrosinase. Although these docking results suggest possible ligand–target interactions, the investigated compounds were not individually validated as enzyme inhibitors using purified-compound biochemical assays. Furthermore, the tentative UHPLC-MS annotations and the absence of direct target-engagement experiments limit mechanistic interpretation. Accordingly, these computational findings should be regarded as hypothesis-generating rather than evidence of experimentally established molecular mechanisms.

**Figure 4:**
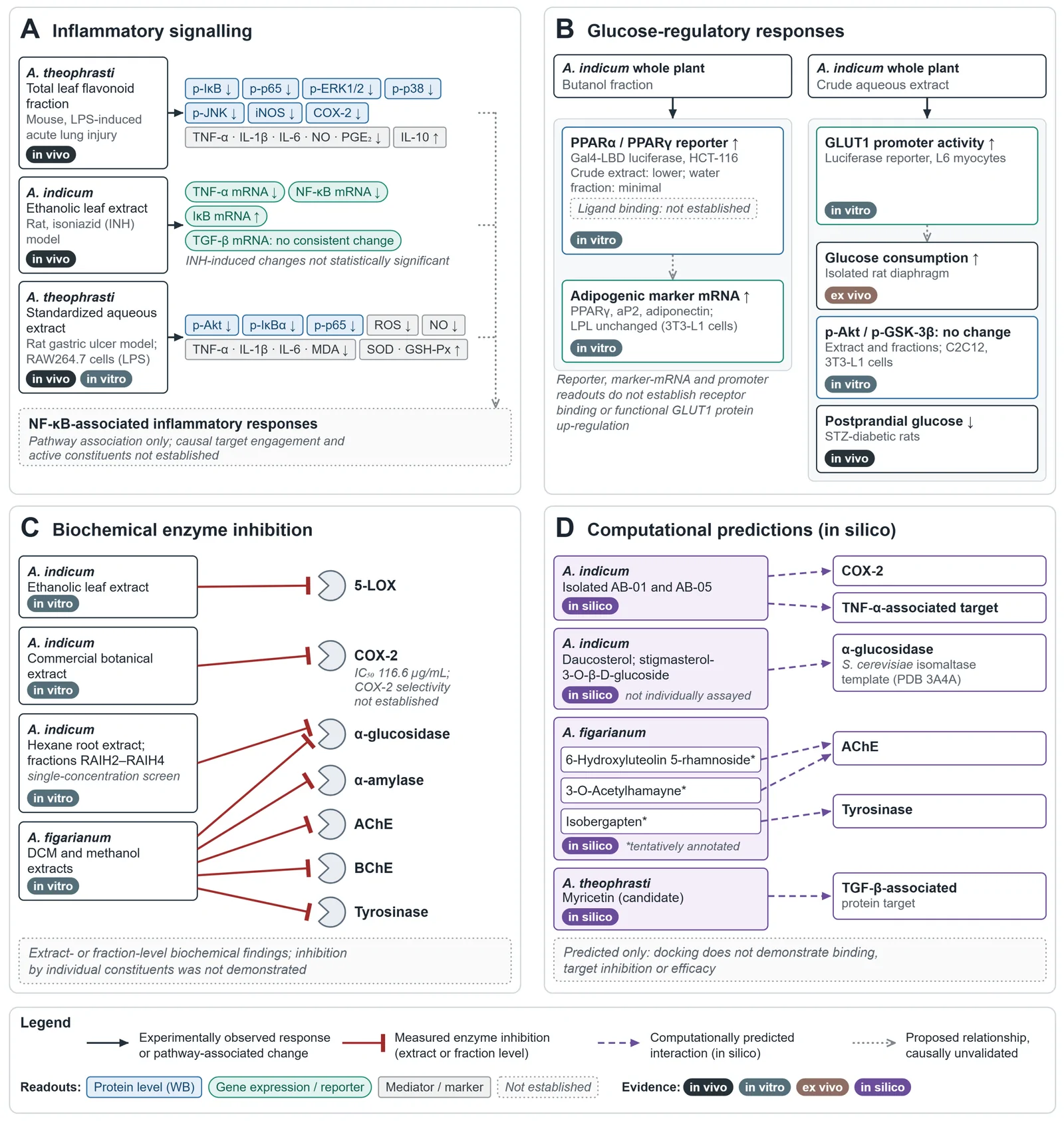
Schematic overview of experimentally observed molecular responses and computationally predicted interactions associated with *Abutilon*-derived preparations. (**A**) Inflammatory signaling responses involving NF-κB/MAPK-associated pathways, inflammatory mediators, and oxidative stress markers. (**B**) Glucose-regulatory responses involving PPARα/PPARγ-associated reporter activation, GLUT1 promoter activity, adipocyte differentiation, and glucose consumption. (**C**) Biochemical inhibition of selected enzymes by botanical extracts or fractions. (**D**) Computationally predicted interactions between phytochemical candidates and molecular targets. Upward (↑) and downward (↓) arrows indicate reported increases and decreases, respectively, in the measured molecular or biological parameters relative to the corresponding experimental controls. An asterisk (*) identifies tentatively annotated phytochemical constituents of *A. figarianum* whose structural identities have not been unequivocally confirmed. Solid arrows indicate experimentally observed responses or pathway-associated changes; blunt-ended lines indicate biochemical enzyme inhibition; dashed arrows indicate in silico predictions; and dotted connections indicate proposed relationships without causal validation. The schematic is based on findings reported in references [7,72,74,76,78,123,124,125,161,182]. The depicted relationships do not establish direct target engagement, causal pathway validation, or clinical efficacy.

The principal experimentally observed pathway-associated responses, biochemical enzyme-inhibitory activities, and computationally predicted molecular interactions reported for *Abutilon*-derived preparations are schematically summarized in Figure 4. The diagram distinguishes experimentally measured responses from proposed ligand–target interactions and highlights the limitations of current mechanistic validation.

To complement this schematic representation, Table 4 critically compares the investigated phytochemical constituents and botanical preparations, their reported biological activities, proposed molecular targets, and the corresponding levels of experimental validation.

### 8.5. Critical Interpretation and Research Priorities

Overall, mechanistic evidence for *Abutilon* remains substantially narrower than the range of reported pharmacological activities. As summarized in Figure 4 and Table 4, the strongest available evidence concerns experimentally observed changes in inflammation-associated signaling, glucose-regulatory responses, and biochemical enzyme inhibition. However, most investigations employ crude extracts or incompletely characterized fractions, and the relationships between individual phytochemical constituents, specific molecular targets, and downstream biological outcomes remain insufficiently validated.

A central limitation is the absence of direct causal validation linking individual phytochemical constituents to specific molecular targets and downstream pharmacological effects. Furthermore, molecular docking predictions cannot substitute for biochemical binding assays, functional target inhibition, or cellular target-engagement experiments. The *A. figarianum* investigation [78] further illustrates this limitation, as extract-level enzyme inhibition and computational ligand–target predictions were reported without direct biochemical validation of the individually proposed inhibitory constituents.

Future studies should prioritize chemically characterized and purified constituents, concentration–response analysis, selective pathway inhibition or genetic validation, quantitative target-engagement assays, and pharmacokinetic assessment of biologically relevant exposure. Integrating these approaches with functional cellular and animal outcomes will be essential for distinguishing direct molecular mechanisms from secondary responses to complex botanical preparations.

## 9. Research Gaps and Future Perspectives

Despite the growing body of phytochemical and pharmacological research on *Abutilon*, several important gaps continue to limit the reproducibility, mechanistic interpretation, and translational relevance of the available evidence. Research remains concentrated predominantly on *A. indicum* and *A. theophrasti*, whereas many other species have received comparatively limited investigation. Future studies should prioritize underexplored species using authenticated botanical material, documented voucher specimens, and consistent taxonomic nomenclature to expand the understanding of phytochemical diversity and identify potentially relevant bioactive constituents.

Methodological heterogeneity represents a major obstacle to meaningful cross-study comparisons. Differences in geographical origin, plant organs, extraction procedures, solvent systems, chemical characterization, administered doses, and experimental models frequently prevent reliable interpretation of relative pharmacological potency. Future investigations should adopt reproducible extraction protocols, validated analytical methods, quantitative chemical fingerprinting, and defined marker compounds. Bioactivity-guided fractionation and direct testing of isolated constituents are particularly important for distinguishing the effects of individual metabolites from those of complex botanical mixtures. In addition, the reporting of concentration–response relationships, appropriate reference controls, and clearly defined experimental endpoints should be strengthened.

Mechanistic investigations should progress beyond phenotypic observations, changes in downstream biomarkers, and molecular docking predictions toward direct experimental validation. Although modulation of NF-κB/MAPK signaling, glucose-regulatory processes, and oxidative stress-related responses has been reported, the causal contributions of individual phytochemicals remain insufficiently established. Future studies should combine chemically characterized preparations with biochemical target-engagement assays, pathway-specific functional experiments, and appropriate cellular and animal models. Particular attention should be given to distinguishing direct molecular interactions from secondary responses associated with complex extracts.

Safety and pharmacokinetic characterization represent additional priorities. Available toxicological studies indicate that safety may vary considerably with the botanical species, plant part, extraction system, formulation, and administered dose. The dose-associated hepatic and genotoxic findings reported for an *A. indicum* seed extract [98] particularly emphasize the need for preparation-specific safety assessment. Future investigations should integrate repeated-dose toxicity, mammalian genotoxicity, reproductive and developmental safety, herb-drug interaction assessment, and pharmacokinetic studies with pharmacologically active exposure ranges. Formulation-based approaches, including phytosomal systems [85], should be evaluated through direct comparative bioavailability and pharmacodynamic studies rather than assuming improved systemic exposure from formulation characteristics alone.

A staged translational pathway from ethnomedicinal knowledge to potential drug candidates is therefore proposed. The initial stage should integrate documented traditional uses with reproducible experimental findings to prioritize promising *Abutilon* species, plant organs, and preparations. Selected materials should then undergo botanical authentication, chemical fingerprinting, marker-based standardization, and bioactivity-guided fractionation to identify chemically defined fractions or candidate constituents. Subsequent development should require reproducible concentration–response relationships, appropriate selectivity assessment, and experimental validation of relevant molecular targets. Progression to advanced preclinical evaluation should depend on demonstrated biological activity at pharmacologically achievable exposures, acceptable pharmacokinetic properties, and preparation-specific safety margins. Candidates failing to meet these criteria should undergo further optimization or be deprioritized. Promising standardized botanical preparations or isolated compounds may subsequently advance through formulation development, comprehensive preclinical safety assessment, and appropriately designed clinical investigations. The small uncontrolled respiratory study involving *A. indicum* [166] illustrates the current limitations of clinical evidence and does not establish therapeutic efficacy. Future randomized controlled trials should employ standardized preparations, appropriate comparators, predefined clinically relevant outcomes, and systematic safety monitoring.

Overall, advancing *Abutilon* research requires a transition from predominantly descriptive phytochemical and pharmacological screening toward integrated, mechanism-informed, and quantitatively standardized investigations. Such an approach would help distinguish preliminary biological activity from pharmacologically meaningful effects and provide a more reliable foundation for evaluating the therapeutic potential of selected *Abutilon*-derived preparations.

## 10. Conclusions

The genus *Abutilon* comprises phytochemically diverse medicinal plants with a long history of ethnomedicinal use and a broad range of experimentally reported biological activities. Among the reviewed species, *A. indicum* and *A. theophrasti* have received the greatest research attention, whereas many other members of the genus remain comparatively underexplored. Flavonoids, phenolic acids, triterpenoids, phytosterols, polysaccharides, and fatty acids represent major reported constituent classes, although the reliability of their identification and quantitative characterization varies considerably among studies.

The available evidence supports preliminary antioxidant, anti-inflammatory, antimicrobial, antidiabetic, hepatoprotective, gastroprotective, and other pharmacological activities, with the strength of evidence differing substantially across experimental models. Most findings originate from biochemical screening, cell-based assays, and animal investigations, whereas controlled clinical evidence remains extremely limited. Mechanistic studies provide additional support for modulation of NF-κB/MAPK signaling, inflammatory mediators, glucose-regulatory processes, and oxidative stress-related responses. Nevertheless, these pathway-associated changes do not independently establish direct molecular target engagement or identify the individual phytochemical constituents responsible for the observed effects.

Investigations involving phytosomal formulations, tablets, and plant-mediated nanoparticles demonstrate opportunities for further pharmaceutical development. However, formulation-associated improvements and nanoparticle-mediated biological effects require careful interpretation because direct pharmacokinetic validation, appropriate formulation controls, and comprehensive safety assessments are frequently lacking. Furthermore, substantial heterogeneity in botanical material, extraction procedures, chemical standardization, dosing regimens, and experimental models limits reliable cross-study comparisons. Available toxicological findings also indicate preparation- and dose-dependent safety concerns, emphasizing that biological activity cannot be equated with therapeutic benefit.

Future research should prioritize authenticated botanical materials, chemically standardized preparations, reproducible concentration–response studies, direct molecular target validation, pharmacokinetic characterization, and comprehensive toxicological evaluation. Promising preparations should advance to controlled clinical investigations only after demonstrating reproducible preclinical activity and acceptable safety profiles. Overall, *Abutilon* represents a potentially valuable source of pharmacologically active natural products, but the current evidence remains insufficient to establish clinical efficacy or standardized therapeutic applications.

## Figures and Tables

**Table 1:** Reported traditional ethnomedicinal uses of selected *Abutilon* species, including plant parts, preparations, and geographical or medical traditions.

Species	Plant Part Used	Preparation	Ethnomedicinal Use	Region/System	References
*A. theophrasti*	Roots, seeds, leaves, aerial parts	Decoction; fresh leaves applied externally with honey or brown sugar; other preparations not consistently specified	Urinary complaints, dysentery, carbuncles, skin conditions, respiratory complaints, rheumatic pain, toothache, hemorrhoids, gonorrhea, otitis media and other reported traditional uses	Traditional Chinese medicine; regional folk practices	[13,44,45,46]
*A. indicum*	Leaves, flowers, seeds, roots, bark	Powder, extract, infusion	Inflammatory conditions, pain, gastrointestinal complaints, wounds	India; Ayurveda	[12,47,48,49]
*A. mauritianum*	Roots, bark, leaves	Infusion, compress	Fever, respiratory and gastrointestinal complaints, dysuria, skin conditions	Africa; traditional medicine	[14]
*A. hirtum*	Roots, leaves, flowers, bark	Decoction, paste	Respiratory complaints, pain, fever, abscesses	Middle East and Africa; folk medicine	[32]
*A. pannosum*	Whole plant	Traditional remedies; preparation not specified	Dysentery, gonorrhea	India; traditional medicine	[52]

Note: Reported ethnomedicinal uses reflect traditional applications and should not be interpreted as evidence of clinically established therapeutic efficacy.

**Table 2:** Reported phytochemical constituents of selected *Abutilon* species, their analytical identification methods, and corresponding extraction systems.

Species and Plant Part	Identified Phytochemical Constituents (Chemical Class)	Analytical and Identification Methods	Extract/Solvent	Reference
*A. theophrasti* Medic., Leaves	Rutin, quercetin 7-O-β-glucopyranoside, kaempferol 3-O-α-L-rhamnopyranosyl-(1→6)-β-D-glucopyranoside, apigenin 7-O-β-diglucoside, poncirin (flavonoid glycosides); luteolin (flavonoid); tiliroside (acylated flavonol glycoside)	LC-DAD and HPLC-ESI-MS/MS	Ethanolic leaf extract	[63]
*A. theophrasti* Medic., Whole plant	Gallic acid, protocatechuic acid, caffeic acid, vanillic acid, syringic acid, p-coumaric acid, ferulic acid, catechin, rutin, quercetin, luteolin, myricetin, naringenin, resveratrol, apiolin, dihydrocoumarin, 2,7-dihydroxy-6-methyl-8-methoxy-1-naphthaldehyde, dibutyl phthalate	RP-HPLC–ESI-QTOF-MS; NMR (reported for selected constituents)	70% ethanolic extract	[64]
*A. theophrasti* Medic., Whole plant	Gallic acid, vanillic acid (phenolic acid); caffeic acid, ferulic acid, protocatechuic acid, syringic acid (phenolic acid); catechin, quercetin (flavonoid); rutin (flavonoid glycoside)	RP-HPLC–UV	70% ethanolic extract	[67]
*A. theophrasti* Medic., Leaves	Gallic acid, protocatechuic acid, chlorogenic acid, vanillic acid, syringic acid, p-hydroxybenzoic acid (phenolic acids)	HPLC; MS; ^1^H and ^13^C NMR	Methanolic extract	[68]
*A. theophrasti* Medic., Seeds	Oleanolic acid (triterpenoid); triacylglycerols, diacylglycerols, free fatty acids (lipids); linoleic acid, palmitic acid, oleic acid, stearic acid (fatty acids)	TLC; HPLC–DAD; GC–MS; FAME analysis	Methanolic extract; petroleum ether fraction	[71]
*A. theophrasti* Medic., Aerial parts	Tentatively annotated flavonoids (including rutin, quercetin, kaempferol and luteolin) and other reported constituents	UPLC–HRMS/MS (tentative annotation); DAD-based rutin quantification	Standardized aqueous extract	[72]
*A. theophrasti* Medic., Aerial parts	Lupenone, lupeol, 24-methylene-3,4-seco-cycloart-4(28)-en-3-oic acid, 24-methylene-3,4-seco-cycloart-4(28)-en-3-methyl ester (triterpenoids); stigmasterol, β-sitosterol (phytosterols); (+)-(1S,4R)-7-hydroxycalamenene (sesquiterpene); hibicuslide C (phenolic compound); isopropyl-β-D-glucopyranoside (glycoside); syringaresinol-4′-O-β-D-glucoside (lignan glycoside); rutin (flavonoid glycoside)	TLC; ESI–MS/EI–MS; ^1^H and ^13^C NMR; 2D NMR; IR	80% methanolic extract; CH_2_Cl_2_, EtOAc and n-BuOH fractions; silica gel, RP-18 and Sephadex LH-20 chromatography	[73]
*A. theophrasti* Medic., Leaves	α-Linolenic acid, hexadecanoic acid/palmitic acid, linoleic acid/9,12-octadecadienoic acid, ethyl linolenate/9,12,15-octadecatrienoic acid ethyl ester (fatty acid)	GC-MS	Petroleum ether extract	[77]
*A. theophrasti* Medic., Aerial parts	(6S,9R)-Roseoside; (6S,9S)-roseoside (C13-norisoprenoid glycosides)	HR-MS; 1D and 2D NMR spectroscopy	Methanolic extract	[81]
*A. theophrasti* Medic., Leaves	Proanthocyanidins (condensed tannins, polyphenols)	Column chromatography; MS (as reported)	Extraction with 90% ethanol under reflux	[82]
*A. theophrasti* Medic., Leaves	Rutin, quercetin (flavonoids)	HPLC–UV	Ionic liquid-based ultrasonic–microwave-assisted extraction ([C4mim]Br)	[83]
*A. indicum* (L.) Sweet, Leaves and flowers	β-Sitosterol (phytosterol); gallic acid (phenolic acid)	RP-HPLC–UV; comparison with authentic standards	Ethanolic extracts	[84]
*A. indicum* (L.) Sweet, aerial parts and roots	Cryptolepine (indoloquinoline alkaloid)	HPTLC–UV densitometry; HPLC–PDA; HRMS	Methanolic extracts	[55]
*A. indicum* (L.) Sweet, Leaves	Eudesmic acid, ferulic acid, caffeic acid (phenolic acids)	IR spectroscopy, ^1^H NMR, ^13^C NMR, MS, chemical tests	Methanolic extract	[69]
*A. indicum* (L.) Sweet, Whole plant	p-Hydroxybenzoic acid, caffeic acid (phenolic acid); p-β-D-glucosyloxybenzoic acid (phenolic glycoside)	Co-TLC; IR; UV; ^1^H and ^13^C NMR; EI–MS; melting point	60% ethanolic extract; chloroform, methanol and n-butanol fractions; silica gel chromatography; acid hydrolysis	[70]
*A. indicum* (L.) Sweet, Aerial parts	5′-Hydroxyhexyl n-hexadecanoate (fatty acid ester); acyl oligoglycoside; β-sitosterol (sterol); lupeol (triterpenoid); ester glycoside.	TLC; ^1^H and ^13^C NMR; MS	Methanolic extract; column chromatography	[74]
*A. indicum* (L.) Sweet, Leaves	Stigmasterol (phytosterol); 3,3′,4′,5,7-pentahydroxyflavone (flavonoid); (3β)-cholest-5-en-3-ol (sterol)	TLC/co-TLC; melting point; UV; IR; ^1^H and ^13^C NMR; EI–MS; elemental analysis	Chloroform extract; silica gel column and preparative TLC	[75]
*A. indicum* (L.) Sweet, Roots	β-Sitosterol, stigmasterol (phytosterol); daucosterol (sterol glycoside; β-sitosterol-3-O-β-D-glucopyranoside); stigmasterol-3-O-β-D-glucoside (sterol glycoside)	TLC; FTIR; ^1^H and ^13^C NMR	Root hexane extract; silica gel chromatography and recrystallization	[76]
*A. indicum* (L.) Sweet, Leaves	Quercetin (flavonoid; 3.65% *w*/*w* of ethanolic leaf extract)	HPTLC quantification using a quercetin reference standard	Ethanolic extract	[85]
*A. indicum* (L.) Sweet; plant part not specified	Alantolactone, isoalantolactone (sesquiterpene lactone)	Co-TLC; melting point; UV, IR, ^1^H NMR, MS	Sequential petroleum ether and alcoholic Soxhlet extraction; chloroform fraction	[86]
*A. indicum* (L.) Sweet, Aerial parts	β-Sitosterol (phytosterol); vanillic acid, p-coumaric acid, caffeic acid, p-hydroxybenzoic acid (phenolic acid); p-β-D-glucosyloxybenzoic acid (phenolic glycoside); fumaric acid (dicarboxylic acid); gluco-vanilloyl glucose (vanillic acid glycoside)	PC; UV; IR; MS; ^1^H NMR; comparison with standards	Petroleum ether and 50% ethanolic extracts; silica gel chromatography; acid hydrolysis and derivative preparation	[87]
*A. indicum* (L.) Sweet, Whole plant	Abutilin A (biphenyl ether); (R)-N-(1′-methoxycarbonyl-2′-phenylethyl)-4-hydroxybenzamide (phenolic acid amide)	^1^H-NMR, ^13^C-NMR, MS, IR	Methanolic extract	[88]
*A. indicum* (L.) Sweet, Leaves	β-Sitosterol and stigmasterol (phytosterol mixture; not individually purified)	TLC; UV–Vis; FTIR; ^1^H and ^13^C NMR; GC–MS	Ethanolic leaf extract (Soxhlet extraction)	[89]
*A. indicum* (L.) Sweet, Flowering tops	Tetradecane and 1,8-cineole (tentatively assigned); caryophyllene oxide; α-pinene, caryophyllene, geraniol, geranyl acetate, eudesmol and farnesol (reported essential-oil constituents)	GLC; TLC; IR; ^1^H NMR; comparison with reference samples	Steam-distilled essential oil from flowering tops; chromatographic fractionation	[90]
*A. figarianum* Webb, whole plant	Tentatively annotated flavonoids, alkaloids, coumarin, phenolic compounds, and fatty-acid derivatives	UHPLC–ESI–QTOF–MS; database-assisted tentative annotation	Methanolic and dichloromethane extracts	[78]
*A. grandiflorum*, Leaves	Kaempferol 3-O-β-(6″-p-coumaroyl)-glucopyranoside, quercetin 3-O-β-glucopyranoside, kaempferol 3-O-β-glucopyranoside, quercetin 3-O-α-L-rhamnopyranosyl-(1→6)-β-D-glucopyranoside, kaempferol 3-O-α-L-rhamnopyranosyl-(1→6)-β-D-glucopyranoside (flavonol glycoside)	UV–Vis; ^1^H and ^13^C NMR; co-chromatography with reference standards	Boiling methanol extraction; CHCl_3_, Et_2_O and n-BuOH partitioning; Sephadex LH-20 and preparative paper chromatography	[66]
*A. bidentatum* (*Hochst*.) *A.Rich.*, Aerial parts	Abubidentin A (oleanane-type triterpenoid ester); 2-hydroxydocosanoic acid (hydroxy fatty acid); stigmasta-22-ene-3β-ol (phytosterol)	TLC; IR; UV; ^1^H and ^13^C NMR; ESI–MS; GC–MS	96% ethanolic extract; petroleum ether fraction; silica gel chromatography; alkaline hydrolysis	[34]
*A. bidentatum* (*Hochst*.) *A.Rich.*, Aerial parts	n-Tetracosane (alkane hydrocarbon); cetyl stearate (fatty acid ester); tetracosyl alcohol (fatty alcohol); cycloartenone (triterpenoid ketone); β-amyrin (pentacyclic triterpenoid); β-sitosterol (phytosterol); alantolactone (sesquiterpene lactone); isoalantolactone (sesquiterpene lactone); cholestane derivative (steroidal compound)	Co-TLC; melting point; IR; ^1^H and ^13^C NMR; MS	Petroleum ether fraction of 95% ethanolic extract; ethanolic fraction; silica gel chromatography	[91]
*A. pannosum* (Forst.f), Leaves	Kaempferol-4′-O-(6″-O-E-p-coumaroyl)-β-D-glucopyranoside (acylated flavonol glycoside)	TLC; UV; IR; ^1^H and ^13^C NMR; MS	Methanolic leaf extract; n-butanol fraction; column chromatography	[92]
*A. pannosum*, *A. hybridum*, *A. hirtum*—aerial parts (leaves and stems)	Tentatively identified phenolic acids (including protocatechuic, ferulic and ellagic acids) and flavonoids (including luteolin, apigenin, quercetin, quercitrin and kaempferol glycosides)	RP-HPLC–DAD; reference-standard comparison	Defatted 80% aqueous methanolic extracts	[93]
*A. hirtum* (Lam.) Sweet, Leaves	Methyl gallate; cuneataside E; bergapten; gallic acid; ellagic acid; epigallocatechin-3-O-gallate; kaempferol-3-O-α-L-rhamnoside; benzyl-1-O-β-D-glucopyranoside; 2-(4-hydroxyphenyl)ethyl-O-β-D-glucopyranoside (phenylethanoid glycoside); β-sitosterol	ESI–MS; ^1^H and ^13^C NMR; melting point; paper chromatography	85% methanolic extract; column chromatography and acid hydrolysis	[94]
*A. hirtum* (Lam.) Sweet, Aerial parts	5,3′-Dihydroxy-3,7,4′-trimethoxyflavone, 5,3′-Dihydroxy-3,6,7,4′-tetramethoxyflavone (flavone); stigmasterol (sterol)	IR; ^1^H and ^13^C NMR; HSQC; HMBC; MS; TLC; silica gel column chromatography	Ethyl acetate extract	[95]

Note: Phytochemical constituents and analytical methods are reported according to the cited studies. Tentative compound annotations are distinguished from structurally characterized constituents and should not be interpreted as unequivocal identifications. Abbreviations are defined in the Abbreviations section.

**Table 4:** Phytochemical-activity-target relationships and mechanistic evidence for *Abutilon* species.

Species/Reference	Constituent or Preparation	Biological Activity	Molecular Target or Pathway	Experimental Evidence and Validation Status
*A. indicum* [7]	Aqueous whole-plant extract; butanol fraction	Glucose-regulatory activity	PPARα/PPARγ; GLUT1 promoter	In vitro reporter assays, adipocyte differentiation, ex vivo glucose consumption, and in vivo glycemic outcomes; direct ligand binding and functional GLUT1 upregulation not established
*A. theophrasti* [72]	Standardized aqueous extract	Gastroprotective and anti-inflammatory	ROS/Akt/IκBα/NF-κB p65	Rat gastric ulcer and RAW264.7 cell models; Western blot, inflammatory and oxidative stress measurements; pathway association demonstrated, causal target engagement unverified
*A. indicum* [74]	Isolated AB-01 and AB-05 constituents	Anti-inflammatory and analgesic	COX-2; TNF-α-associated molecular target	In vivo phenotypic activity and in silico docking; no purified-compound biochemical target-inhibition confirmation
*A. indicum* [76]	Hexane root extract and fractions; isolated sterols and sterol glycosides	α-Glucosidase inhibition	α-Glucosidase	In vitro fraction-level inhibition and in silico docking; isolated compounds not individually assayed; yeast enzyme model
*A. figarianum* [78]	Tentatively annotated 6-hydroxyluteolin 5-rhamnoside, 3-O-acetylhamayne, and isobergapten	Predicted cholinesterase and tyrosinase inhibition	AChE; tyrosinase	Extract-level biochemical inhibition and compound-level in silico docking; no purified-compound validation
*A. indicum* [123]	Ethanolic leaf extract	Anti-inflammatory; antiproliferative	5-LOX; Apaf-1-associated protein network	In vitro 5-LOX inhibition and A549 viability assay; Apaf-1 network computationally inferred, not experimentally validated
*A. indicum* [124]	Ethanolic leaf extract	Modulation of inflammatory responses	TNF-α, NF-κB, IκB gene expression	In vivo isoniazid-induced rat model; gene-expression measurements; protein activation and pathway causality not established
*A. theophrasti* [125]	Total leaf flavonoid fraction	Anti-inflammatory; protection against acute lung injury	NF-κB/MAPK; iNOS; COX-2	In vivo LPS-induced mouse model; Western blot and cytokine assays; individual active constituents and direct binding unverified
*A. theophrasti* [161]	Myricetin (computational candidate)	Proposed anticancer potential	TGF-β-associated protein target	In silico docking (−8.7 kcal/mol); no experimental target inhibition or anticancer validation
*A. indicum* [182]	Commercial botanical extract	COX-2 inhibition	COX-2	In vitro enzyme assay (reported IC_50_ 116.6 µg/mL); limited chemical characterization and no demonstrated COX-2 selectivity

Note: The table distinguishes experimentally observed biological responses, pathway-associated changes, biochemical enzyme inhibition, and computationally predicted ligand-target interactions. Evidence obtained with crude extracts or fractions should not be attributed to individual phytochemical constituents without compound-specific validation. Molecular docking scores represent computational predictions and do not establish direct binding or functional target inhibition. Abbreviations are defined in the Abbreviations section.

## Data Availability

No new data were created or analyzed in this study. Data sharing is not applicable to this article.

## Supplementary Materials

The following supporting information can be downloaded at: https://www.mdpi.com/article/10.3390/ijms27219768/s1.

## Abbreviations

The following abbreviations are used in this manuscript:

ABTS	2,2′-Azino-bis(3-ethylbenzothiazoline-6-sulfonic acid)
AChE	Acetylcholinesterase
ALP	Alkaline phosphatase
BSA	Bovine serum albumin
CAT	Catalase
COX-2	Cyclooxygenase-2
DAD	Diode-array detector
DPPH	2,2-Diphenyl-1-picrylhydrazyl
EI-MS	Electron ionization mass spectrometry
ESI-MS	Electrospray ionization mass spectrometry
FAME	Fatty acid methyl esters
FEF_25–75%_	Forced expiratory flow at 25–75% of forced vital capacity
FEV_1_	Forced expiratory volume in one second
FID	Flame ionization detector
FRAP	Ferric reducing antioxidant power
FTIR	Fourier-transform infrared spectroscopy
FVC	Forced vital capacity
GC–MS	Gas chromatography–mass spectrometry
GLC	Gas–liquid chromatography
GLUT1	Glucose transporter 1
GSH-Px	Glutathione peroxidase
HPLC	High-performance liquid chromatography
HRMS	High-resolution mass spectrometry
IC_50_	Half-maximal inhibitory concentration
IκB	Inhibitor of nuclear factor kappa B
IL-1β	Interleukin-1 beta
IL-4	Interleukin-4
IL-6	Interleukin-6
IL-UMAE	Ionic liquid-based ultrasonic–microwave-assisted extraction
iNOS	Inducible nitric oxide synthase
IR	Infrared spectroscopy
LC_50_	Median lethal concentration
LC–MS/MS	Liquid chromatography–tandem mass spectrometry
LOD	Limit of detection
LOQ	Limit of quantification
LPS	Lipopolysaccharide
MAPK	Mitogen-activated protein kinase
MBC	Minimum bactericidal concentration
MDA	Malondialdehyde
MES	Maximal electroshock
MIC	Minimum inhibitory concentration
MVV	Maximum voluntary ventilation
NF-κB	Nuclear factor kappa B
NMR	Nuclear magnetic resonance
NO	Nitric oxide
PC	Paper chromatography
POWO	Plants of the World Online
PTZ	Pentylenetetrazole
QTOF-MS	Quadrupole time-of-flight mass spectrometry
ROS	Reactive oxygen species
RP-HPLC	Reversed-phase high-performance liquid chromatography
SGOT	Serum glutamic-oxaloacetic transaminase
SGPT	Serum glutamic-pyruvic transaminase
SOD	Superoxide dismutase
TAC	Total antioxidant capacity
TGF-β	Transforming growth factor beta
TLC	Thin-layer chromatography
TNF-α	Tumor necrosis factor alpha
UPLC	Ultra-performance liquid chromatography
UV	Ultraviolet
UV–Vis	Ultraviolet–visible spectroscopy
WFO	World Flora Online
