# Supplementary material for: From Ethnomedicine to Pharmacological Evidence: Phytochemistry, Biological Activities, and Therapeutic Potential of the Genus Abutilon (Malvaceae)
Source: Int J Mol Sci. 2026 Oct 30;27(21):9768. doi: 10.3390/ijms27219768 (PMC13762928; doi:10.3390/ijms27219768)
Supplement: Supplementary file 1 [file ijms-27-09768-s001.zip › ijms-4605291-supplementary.pdf]

**Supplementary Table S1.** Additional pharmacological studies on *Abutilon* species (excluding studies selected for main-text Table 3).

Evidence categories describe experimental context and do not constitute a formal risk-of-bias score.

| Species [Ref.] | Plant material / preparation | Experimental model | Dose / concentration | Main findings | Evidence category |
|---|---|---|---|---|---|
| **Antioxidant Activity** | | | | | |
| *A. indicum* [100] | Crude saponin-enriched leaf extract | DPPH radical-scavenging assay | 0.25–2.5 mg/mL | DPPH inhibition increased from 15.7% to 96.17%; chemical composition incompletely characterized | Chemical assay |
| *A. indicum* [102] | Hydroethanolic leaf extract | DPPH and related antioxidant assays | 5–300 μg/mL | Reported DPPH $IC_{50}$ = 89.49 ± 1.23 μg/mL; inconsistencies in quantitative reporting limit interpretation | Chemical assay |
| **Anti-inflammatory Activity** | | | | | |
| *A. indicum* [119] | Ethanolic leaf extract | Carrageenan-induced paw edema in rats | 300 mg/kg, p.o. | 64.04% edema inhibition | *In vivo* |
| *A. hirtum* [126] | Ethanolic leaf extract and chloroform fraction | Carrageenan-induced paw edema in rats | 300 mg/kg | 50.82% edema inhibition after 5 h; reference-drug reporting inconsistent | *In vivo* |
| **Antimicrobial Activity** | | | | | |
| *A. indicum* [100] | Crude saponin-enriched leaf fraction | *S. aureus, E. coli* | MIC = 1.11 mg/mL | Inhibition zones of 17 and 15 mm, respectively; active constituents not individually established | *In vitro* |
| *A. indicum* [57] | Electrospun PVA nanofibrous mats containing leaf extract | *E. coli,* agar diffusion assay | Polymer-to-extract formulations 90:10 to 50:50 | Inhibition zones 10–23 mm for extract-containing formulations; unloaded PVA inactive. MIC, release kinetics, and in vivo efficacy not established. | *In vitro;* formulation-level |
| *A. indicum* [127] | Ethanolic leaf extract | Five periodontal bacterial species | 0.2–100 mg/mL | Concentration-dependent antibacterial activity | *In vitro* |
| *A. theophrasti* [134] | Phenolic leaf extract | *E. coli and S. aureus,* including clinical isolates | MIC = 1.21–4.93 mg/mL | Antibacterial activity against 24 tested strains | *In vitro* |
| *A. pannosum* [140] | Aqueous, methanolic, petroleum ether, and isopropanolic leaf extracts | Agar-well diffusion; 11 bacterial strains | 10–250 μg/mL | Inhibition zones approximately 9–14 mm against selected susceptible bacteria; MIC not determined | *In vitro* |
| *A. pannosum* [141] | Methanolic leaf extract | Agar-well diffusion against eight Gram-positive and Gram-negative bacterial species | 25 mg/mL; 0.1 mL/well | Inhibition zones of 17.1–23.5 mm; MIC not determined | *In vitro* |
| **Hepatoprotective Activity** | | | | | |
| *A. indicum* [146] | Aqueous leaf extract | $CCl_4$- and paracetamol-induced hepatotoxicity in Wistar rats | 100, 200 mg/kg, p.o. | Reduced SGOT, SGPT, ALP and bilirubin; partially restored hepatic GSH | *In vivo* |
| *A. indicum* [147] | 70% ethanolic flower extract | $CCl_4$-induced hepatotoxicity in rats | 100, 250, 500 mg/kg | Reduced serum liver injury markers and bilirubin | *In vivo* |
| *A. indicum* [148] | Aqueous root extract | Lead acetate-induced hepatotoxicity in Wistar rats | 300, 500 mg/kg | Reduced MDA; increased SOD, CAT and GPx | *In vivo* |
| *A. hirtum* [150] | Aqueous leaf decoction | $CCl_4$-induced hepatotoxicity in rats | 100, 200 mg/kg | Reduced liver injury markers; histological improvement reported | *In vivo* |

| Species [Ref.] | Plant material / preparation | Experimental model | Dose / concentration | Main findings | Evidence category |
|---|---|---|---|---|---|
| **Antidiabetic Activity** | | | | | |
| *A. indicum* [152] | Chloroform fraction | STZ-induced diabetic rats | 50 mg/kg, p.o., 21 days | Blood glucose decreased from 373.6 ± 4.9 to 195.1 ± 3.7 mg/dL | *In vivo* |
| *A. indicum* [154] | 50:50 ethanol–water leaf extract | Antiglycation and fructosamine-formation assays | 25 μg/mL for AGE inhibition | 60% inhibition of AGE formation; 71% inhibition of fructosamine formation after 3 days (reported without detailed quantitative supporting data) | *In vitro*; biochemical |
| *A. indicum* [156] | Petroleum ether, benzene, alcoholic and aqueous leaf extracts | Healthy rats | 400 mg/kg | Blood glucose reductions of 34.69%, 46.34%, 30.30% and 53.55%, respectively | *In vivo* |
| *A. indicum* [157] | Ethanolic and aqueous leaf extracts | Normal rats | 400 mg/kg | Significant hypoglycemic effects reported | *In vivo* |
| *A. hirtum* [126] | Ethanolic leaf extract and solvent-derived fractions | STZ-induced diabetic rats | 300 mg/kg | Glucose-lowering activity; crude polysaccharide fraction showed the greatest effect | *In vivo* |
| *A. hirtum* [158] | Leaf extract | α-Amylase inhibition assay | $IC_{50}$ = 119.48 μg/mL | Lower inhibitory potency than acarbose ($IC_{50}$ = 12.53 μg/mL) | *In vitro*; biochemical |
| **Antitumor Activity** | | | | | |
| *A. indicum* [123] | Ethanolic leaf extract | A549 lung cancer cells; XTT assay | $IC_{50}$ = 85.2 μg/mL | 72.1% growth inhibition at 200 μg/mL; cisplatin $IC_{50}$ = 24.9 μg/mL | *In vitro* |
| *A. indicum* [159] | Aqueous leaf-extract-mediated iron oxide nanoparticles (Ai-FeO NPs; 10–95 nm) | MDA-MB-231 breast cancer cells; MTT, AO/EB and DAPI assays | 10–100 μg/mL; exposure duration inconsistently reported (24/48 h) | Cell viability decreased from 96% to 13%; apoptosis-associated morphological changes; conflicting $IC_{50}$ values prevent reliable potency comparison | *In vitro*; nanomaterial |
| *A. hirtum* [116] | Aqueous extract | MCF-7 breast cancer cells; MTT assay | $IC_{50}$ = 368.7 μg/mL | 43.71% inhibition at 300 μg/mL | *In vitro* |
| *A. theophrasti* [161] | Myricetin; computational screening | Gastric cancer-associated TGF-β signaling protein target | Docking score −8.7 kcal/mol | Predicted molecular interaction; no experimental validation | *In silico* |
| **Neurotropic and Neuroprotective Activities** | | | | | |
| *A. indicum* [163] | Crude methanolic leaf-and-bark extract | Swiss albino mice; elevated plus maze, light–dark exploration, hole-board, forced swim and tail suspension tests | 30, 50, 100 mg/kg, i.p. | Dose-associated anxiolytic- and antidepressant-like behavioral responses, with the greatest reported effects at 100 mg/kg | *In vivo* |
| **Analgesic Activity** | | | | | |
| *A. indicum* [168] | Petroleum ether, methanolic, ethanolic and aqueous root extracts | Acetic acid-induced writhing, tail-flick and tail-immersion assays in Swiss albino mice | 200, 400 mg/kg, p.o. | At 400 mg/kg, writhing inhibition was 30.78% (petroleum ether), 25.00% (aqueous) and 14.11% (ethanolic); methanolic extract showed no significant activity; petroleum ether extract produced the strongest overall response | *In vivo* |
| **Diuretic Activity** | | | | | |
| *A. indicum* [169] | Aqueous seed extract | Rat diuretic model; urinary volume | 200, 400 mg/kg | Increased urine output and $Na^+$ excretion; no | *In vivo* |

| Species [Ref.] | Plant material / preparation | Experimental model | Dose / concentration | Main findings | Evidence category |
|---|---|---|---|---|---|
| | | and electrolyte measurements | | significant corresponding increase in $K^+$ excretion reported | |
| *A. indicum* [170] | Ethanolic and chloroform whole-plant extracts | Diuretic assessment in albino rats; 0.75% ethylene glycol-induced urolithiasis for 28 days | 400 mg/kg | Ethanolic extract showed reported diuretic and antiurolithiatic activity; urinary volume, $Na^+$, $K^+$, $Cl^-$ and renal biochemical parameters were evaluated | *In vivo* |
| **Additional Cytotoxicity Evidence** | | | | | |
| *A. indicum* [172] | Leaf-mediated MnO nanoparticles | HeLa cells; MTT assay, 24 h | 1–120 μg/mL | Concentration-dependent cytotoxicity; more than 50% cell death reported at 120 μg/mL | *In vitro;* nanomaterial |
| *A. indicum* [173] | Flavonoid-rich leaf extract | SK-LU-1, HepG2 and MKN7 cells; SRB assay | $IC_{50}$ = 148.29 ± 4.55, 164.39 ± 6.94 and 164.76 ± 7.73 μg/mL, respectively | Cytotoxic activity against three cancer cell lines; selectivity not established | *In vitro* |
| *A. indicum* [174] | Chloroform fraction of methanolic extract | Brine shrimp lethality assay | $LC_{50}$ = 1.51 μg/mL | Marked lethality in a preliminary toxicity-screening model | *In vitro*; toxicity screening |
| *A. fruticosum* [175] | Ethyl acetate fraction of 70% ethanolic aerial-part extract | H1299 and HepG2 cells; SRB assay, 48 h | $IC_{50}$ = 63.62 ± 0.05 and 102 ± 0.6 μg/mL, respectively | Greater cytotoxicity than corresponding butanol fraction; selectivity not established | *In vitro* |
| *A. pannosum* [176] | Chloroform fraction | MCF-7 cells; viability, cell-cycle and apoptosis assays | $IC_{50}$ = 50 μg/mL | Reduced cell proliferation, G1-phase arrest and apoptosis-associated changes | *In vitro* |
| *A. hirtum* [178] | Whole-plant aqueous extract-mediated AgNPs (15–30 nm) | HepG2 cells; MTT assay, 24 h | 6–200 μg/mL; reported $IC_{50}$ = 50.78 ± 3.72 μM | Greater cytotoxicity than parent extract; inconsistent $IC_{50}$ units limit interpretation | *In vitro*; nanomaterial |
| **Gastroprotective and Antiulcer Activities** | | | | | |
| *A. indicum* [108] | Ethanolic flower extract | Ethanol-induced gastric ulcer model | 250, 500 mg/kg; | Reduced ulcer index and gastric acidity; increased gastric pH | *In vivo* |
| *A. indicum* [179] | Methanolic leaf extract | Pylorus-ligation and ethanol-induced gastric ulcer models | 250, 500 mg/kg; | Reduced gastric juice volume, acidity and ulcer formation | *In vivo* |
| **Enzyme-Inhibiting Activity** | | | | | |
| *A. indicum* [183] | Alcoholic extract; precise plant material requires confirmation | Pepsin–hemoglobin enzyme inhibition assay | 1 mg/mL; pepstatin 100 μg/mL | Pepsin inhibition 40.81%, compared with 93.87% for pepstatin; HIV protease inhibition not demonstrated | *In vitro*; biochemical |
| **Aphrodisiac Activity** | | | | | |
| *A. pannosum* [185] | Methanolic stem-bark extract | Male rats; sexual-behavior tests, sperm parameters and serum testosterone | 400 mg/kg daily for 5, 10 and 15 days | Reduced mount and intromission latency and post-ejaculatory interval; increased selected copulatory measures, sperm viability and motility, and testosterone; total sperm count increase was not significant | *In vivo* |
| **Other Reported Pharmacological Activities** | | | | | |
| *A. theophrasti* [186] | Methanolic stem extract | H. contortus egg-hatch and L3 larval-motility assays | 31.25–500 mg/mL | At 500 mg/mL, egg-hatch inhibition 74.39%; larval-motility inhibition 79.79% | *In vitro* |
| *A. indicum* [187] | Aqueous and ethanolic leaf | Humoral and cellular immune- | 200 mg/kg (ethanolic); 400 | Extract-dependent immune responses; phagocytic | *In vivo* |

| Species [Ref.] | Plant material / preparation | Experimental model | Dose / concentration | Main findings | Evidence category |
| --- | --- | --- | --- | --- | --- |
| | extracts | response models | mg/kg (aqueous) | index 0.076 (ethanolic), 0.017 (aqueous), 0.029 (control) | |
| *A. indicum* [188] | Methanolic and aqueous leaf extracts | Castor oil-induced diarrhea; $PGE_2$-induced enteropooling; intestinal transit | 500 mg/kg | Fecal output reduced to 10.25 ± 0.22 and 15.38 ± 0.53 g/kg versus 23.52 ± 0.34 g/kg in controls | *In vivo* |
| *A. indicum* [189] | 50% methanolic fruit extract | Ovariectomized rats; uterotropic and peroxidase assays | 100, 200, 500 mg/kg | At 500 mg/kg, estradiol-stimulated uterine weight decreased from 77.8 to 33.2 mg/100 g | *In vivo* |
| *A. indicum* [183] | Methanolic leaf preparation | M. tuberculosis H37Rv; Microplate Alamar Blue Assay | 12.5–100 μg/mL | Growth inhibition reported; limited methodological validation | *In vitro* |
| *A. indicum* [190] | 5% extract-containing ointment | Excision and incision wound models in Wistar rats | 5% w/w, topical | Wound area decreased to 10 ± 3.11 on day 14 and zero by day 16 | *In vivo* |
| *A. indicum* [191] | Methanolic leaf extract | E. carinatus venom-associated enzyme assays | 250 μg/mL | Inhibited several venom-associated enzymes; systemic venom neutralization not demonstrated | *In vitro* |
| *A. pannosum* [114] | Ethanolic extract | G. lamblia trophozoites; Vero-cell cytotoxicity | 500 μg/mL, 72 h | 79% inhibition; antigiardial $IC_{50}$ 89.7 μg/mL; Vero-cell $IC_{50}$ 279.94 μg/mL | *In vitro* |
| *A. hirtum* [193] | Chloroform leaf fraction | L. donovani intracellular amastigote screening | 20 μg/mL | 78% initial inhibition; follow-up $IC_{50}$ >20 μg/mL | *In vitro* |

Note: Studies are grouped by pharmacological domain. Some references appear in more than one domain because they report distinct experimental endpoints. Differences in models, extraction systems, doses, and controls preclude direct cross-study potency comparisons.
